# Supplementary material for: STEED: A data mining tool for automated extraction of experimental parameters and risk of bias items from in vivo publications
Source: PLoS One. 2024 Nov 26;19(11):e0311358. doi: 10.1371/journal.pone.0311358 (PMC11594395; doi:10.1371/journal.pone.0311358)
Supplement: S1 File — (PDF) [file pone.0311358.s001.pdf]

Supplementary reference list

Training set

<u>Number of records</u>: 45

<u>Topic</u>: Amyotrophic lateral sclerosis

[1] I. Allodi, R. Montañana-Rosell, R. Selvan, P. Löw, and O. Kiehn, Locomotor deficits in a mouse model of ALS are paralleled by loss of V1-interneuron connections onto fast motor neurons. Nat Commun 12 (2021) 3251.

[2] S. Anzilotti, V. Valsecchi, P. Brancaccio, N. Guida, G. Laudati, V. Tedeschi, T. Petrozziello, F. Frecentese, E. Magli, B. Hassler, O. Cuomo, L. Formisano, A. Secondo, L. Annunziato, and G. Pignataro, Prolonged NCX activation prevents SOD1 accumulation, reduces neuroinflammation, ameliorates motor behavior and prolongs survival in a ALS mouse model. Neurobiol Dis 159 (2021) 105480.

[3] S. Apolloni, P. Fabbrizio, S. Amadio, G. Napoli, M. Freschi, F. Sironi, P. Pevarello, P. Tarroni, C. Liberati, C. Bendotti, and C. Volonté, Novel P2X7 Antagonist Ameliorates the Early Phase of ALS Disease and Decreases Inflammation and Autophagy in SOD1-G93A Mouse Model. Int J Mol Sci 22 (2021).

[4] A. Arnoux, E. Ayme-Dietrich, S. Dieterle, M.A. Goy, S. Schann, M. Frauli, L. Monassier, and L. Dupuis, Evaluation of a 5-HT(2B) receptor agonist in a murine model of amyotrophic lateral sclerosis. Sci Rep 11 (2021) 23582.

[5] D. Bayer, S. Antonucci, H.P. Müller, R. Saad, L. Dupuis, V. Rasche, T.M. Böckers, A.C. Ludolph, J. Kassubek, and F. Roselli, Disruption of orbitofrontal-hypothalamic projections in a murine ALS model and in human patients. Transl Neurodegener 10 (2021) 17.

[6] T. Burg, E. Rossaert, M. Moisse, P. Van Damme, and L. Van Den Bosch, Histone Deacetylase Inhibition Regulates Lipid Homeostasis in a Mouse Model of Amyotrophic Lateral Sclerosis. Int J Mol Sci 22 (2021).

[7] I. Casola, B.M. Scicchitano, E. Lepore, S. Mandillo, E. Golini, C. Nicoletti, L. Barberi, G. Dobrowolny, and A. Musarò, Circulating myomiRs in Muscle Denervation: From Surgical to ALS Pathological Condition. Cells 10 (2021).

[8] L. Chen, R. Na, K. Danae McLane, C.S. Thompson, J. Gao, X. Wang, and Q. Ran, Overexpression of ferroptosis defense enzyme Gpx4 retards motor neuron disease of SOD1G93A mice. Sci Rep 11 (2021) 12890.

[9] K. Cieminski, D.J. Flis, K. Dzik, J.J. Kaczor, E. Czyrko, M. Halon-Golabek, M.R. Wieckowski, J. Antosiewicz, and W. Ziolkowski, Swim training affects Akt signaling and ameliorates loss of skeletal muscle mass in a mouse model of amyotrophic lateral sclerosis. Sci Rep 11 (2021) 20899.

[10] G. Cocozza, S. Garofalo, M. Morotti, G. Chece, A. Grimaldi, M. Lecce, F. Scavizzi, R. Menghini, V. Casagrande, M. Federici, M. Raspa, H. Wulff, and C. Limatola, The feeding behaviour of Amyotrophic Lateral Sclerosis mouse models is modulated by the Ca(2+) -activated K(Ca) 3.1 channels. Br J Pharmacol 178 (2021) 4891-4906.

[11] K.P. Dzik, D.J. Flis, Z.K. Bytowska, M.J. Karnia, W. Ziolkowski, and J.J. Kaczor, Swim Training Ameliorates Hyperlocomotion of ALS Mice and Increases Glutathione Peroxidase Activity in the Spinal Cord. Int J Mol Sci 22 (2021).

[12] P. Fabbrizio, J. D'Agostino, C. Margotta, G. Mella, N. Panini, L. Pasetto, E. Sammali, F. Raggi, G. Sorarù, V. Bonetto, C. Bendotti, and G. Nardo, Contingent intramuscular boosting of P2XR7 axis improves motor function in transgenic ALS mice. Cell Mol Life Sci 79 (2021) 7.

[13] L.C. Fernández-Beltrán, J.M. Godoy-Corchuelo, M. Losa-Fontangordo, D. Williams, J. Matias-Guiu, and S. Corrochano, A Transcriptomic Meta-Analysis Shows Lipid Metabolism Dysregulation as an Early Pathological Mechanism in the Spinal Cord of SOD1 Mice. Int J Mol Sci 22 (2021).

[14] A. Ferrer-Donato, A. Contreras, L.M. Frago, J.A. Chowen, and C.M. Fernandez-Martos, Alterations in Leptin Signaling in Amyotrophic Lateral Sclerosis (ALS). Int J Mol Sci 22 (2021).

[15] S. Garbuzova-Davis, K.J. Boccio, A. Llauget, R. Shell, S. Hailu, H. Mustafa, J. Ehrhart, P.R. Sanberg, S.H. Appel, and C.V. Borlongan, Beneficial Effects of Transplanted Human Bone Marrow Endothelial Progenitors on Functional and Cellular Components of Blood-Spinal Cord Barrier in ALS Mice. eNeuro 8 (2021).

[16] Á. Gento-Caro, E. Vilches-Herrando, F. Portillo, D. González-Forero, and B. Moreno-López, Targeting autotaxin impacts disease advance in the SOD1-G93A mouse model of amyotrophic lateral sclerosis. Brain Pathol 32 (2022) e13022.

[17] M. Gómez-Almería, S. Burgaz, C. Costas-Insua, C. Rodríguez-Cueto, I. Santos-García, I. Rodríguez-Crespo, C. García, M. Guzmán, E. de Lago, and J. Fernández-Ruiz, BiP Heterozigosity Aggravates Pathological Deterioration in Experimental Amyotrophic Lateral Sclerosis. Int J Mol Sci 22 (2021).

[18] F. Gouel, K. Timmerman, P. Gosset, C. Raoul, M. Dutheil, A. Jonneaux, G. Garçon, C. Moreau, V. Danel-Brunaud, J. Duce, T. Burnouf, J.C. Devedjian, and D. Devos, Whole and fractionated human platelet lysate biomaterials-based biotherapy induces strong neuroprotection in experimental models of amyotrophic lateral sclerosis. Biomaterials 280 (2022) 121311.

[19] A. Keerie, H. Brown-Wright, I. Kirkland, A. Grierson, J.J.P. Alix, C. Holscher, and R.J. Mead, The GLP-1 receptor agonist, liraglutide, fails to slow disease progression in SOD1(G93A) and TDP-43(Q331K) transgenic mouse models of ALS. Sci Rep 11 (2021) 17027.

[20] I. Keskin, E. Ekhtiari Bidhendi, M. Marklund, P.M. Andersen, T. Brännström, S.L. Marklund, and U. Nordström, Peripheral administration of SOD1 aggregates does not transmit pathogenic aggregation to the CNS of SOD1 transgenic mice. Acta Neuropathol Commun 9 (2021) 111.

[21] V. La Cognata, E. Golini, R. Iemmolo, S. Balletta, G. Morello, C. De Rosa, A. Villari, S. Marinelli, V. Vacca, G. Bonaventura, P. Dell'Albani, E. Aronica, F. Mammano, S. Mandillo, and S. Cavallaro, CXCR2 increases in ALS cortical neurons and its inhibition prevents motor neuron degeneration in vitro and improves neuromuscular function in SOD1G93A mice. Neurobiol Dis 160 (2021) 105538.

[22] P.A. Leermakers, M. Skov, A. Riisager, O.B. Nielsen, and T.H. Pedersen, Alterations in fast-twitch muscle membrane conductance regulation do not explain decreased muscle function of SOD1(G93A) rats. Muscle Nerve 64 (2021) 755-764.

[23] X. Li, C. Chen, X. Zhan, B. Li, Z. Zhang, S. Li, Y. Xie, X. Song, Y. Shen, J. Liu, P. Liu, G.P. Liu, and X. Yang, R13 preserves motor performance in SOD1(G93A) mice by improving mitochondrial function. Theranostics 11 (2021) 7294-7307.

[24] J.S. Lum, M.L. Brown, N.E. Farrawell, L. McAlary, D. Ly, C.G. Chisholm, J. Snow, K.L. Vine, T. Karl, F. Kreilaus, L.E. McInnes, S. Nikseresht, P.S. Donnelly, P.J. Crouch, and J.J. Yerbury, CuATSM improves motor function and extends survival but is not tolerated at a high dose in SOD1(G93A) mice with a C57BL/6 background. Sci Rep 11 (2021) 19392.

[25] M. MacLean, J. Juranek, S. Cuddapah, R. López-Díez, H.H. Ruiz, J. Hu, L. Frye, H. Li, P.F. Gugger, and A.M. Schmidt, Microglia RAGE exacerbates the progression of neurodegeneration within the SOD1(G93A) murine model of amyotrophic lateral sclerosis in a sex-dependent manner. J Neuroinflammation 18 (2021) 139.

[26] Y.F. Malada Edelstein, Y. Solomonov, N. Hadad, L. Alfahel, A. Israelson, and R. Levy, Early upregulation of cytosolic phospholipase A(2)α in motor neurons is induced by misfolded SOD1 in a mouse model of amyotrophic lateral sclerosis. J Neuroinflammation 18 (2021) 274.

[27] R. Massopust, D. Juros, D. Shapiro, M. Lopes, S.M. Haldar, T. Taetzsch, and G. Valdez, KLF15 overexpression in myocytes fails to ameliorate ALS-related pathology or extend the lifespan of SOD1G93A mice. Neurobiol Dis 162 (2022) 105583.

[28] T.S. McDonald, V. Kumar, J.N. Fung, T.M. Woodruff, and J.D. Lee, Glucose clearance and uptake is increased in the SOD1(G93A) mouse model of amyotrophic lateral sclerosis through an insulin-independent mechanism. Faseb j 35 (2021) e21707.

[29] I. Méndez-López, F.J. Sancho-Bielsa, T. Engel, A.G. García, and J.F. Padín, Progressive Mitochondrial SOD1(G93A) Accumulation Causes Severe Structural, Metabolic and Functional Aberrations through OPA1 Down-Regulation in a Mouse Model of Amyotrophic Lateral Sclerosis. Int J Mol Sci 22 (2021).

[30] M. Milani, E. Mammarella, S. Rossi, C. Miele, S. Lattante, M. Sabatelli, M. Cozzolino, N. D'Ambrosi, and S. Apolloni, Targeting S100A4 with niclosamide attenuates inflammatory and profibrotic pathways in models of amyotrophic lateral sclerosis. J Neuroinflammation 18 (2021) 132.

[31] E. Minj, S. Upadhayay, and S. Mehan, Nrf2/HO-1 Signaling Activator Acetyl-11-keto-beta Boswellic Acid (AKBA)-Mediated Neuroprotection in Methyl Mercury-Induced Experimental Model of ALS. Neurochem Res 46 (2021) 2867-2884.

[32] N. Ohashi, T. Terashima, M. Katagi, Y. Nakae, J. Okano, Y. Suzuki, and H. Kojima, GLT1 gene delivery based on bone marrow-derived cells ameliorates motor function and survival in a mouse model of ALS. Sci Rep 11 (2021) 12803.

[33] I. Orienti, M. Armida, G. Dobrowolny, R. Pepponi, G. Sollazzini, A. Pezzola, I. Casola, A. Musarò, P. Popoli, and R.L. Potenza, Fenretinide Beneficial Effects on Amyotrophic Lateral Sclerosis-associated SOD1(G93A) Mutant Protein Toxicity: In Vitro and In Vivo Evidences. Neuroscience 473 (2021) 1-12.

[34] L. Pasetto, S. Callegaro, A. Corbelli, F. Fiordaliso, D. Ferrara, L. Brunelli, G. Sestito, R. Pastorelli, E. Bianchi, M. Cretich, M. Chiari, C. Potrich, C. Moglia, M. Corbo, G. Sorarù, C. Lunetta, A. Calvo, A. Chiò, G. Mora, M. Pennuto, A. Quattrone, F. Rinaldi, V.G. D'Agostino, M. Basso, and V. Bonetto, Decoding distinctive features of plasma extracellular vesicles in amyotrophic lateral sclerosis. Mol Neurodegener 16 (2021) 52.

[35] M. Peric, L. Nikolic, P.R. Andjus, and D. Bataveljic, Dysfunction of oligodendrocyte inwardly rectifying potassium channel in a rat model of amyotrophic lateral sclerosis. Eur J Neurosci 54 (2021) 6339-6354.

[36] J. Post, A. Schaffrath, I. Gering, S. Hartwig, S. Lehr, N.J. Shah, K.J. Langen, D. Willbold, J. Kutzsche, and A. Willuweit, Oral Treatment with RD2RD2 Impedes Development of Motoric Phenotype and Delays Symptom Onset in SOD1(G93A) Transgenic Mice. Int J Mol Sci 22 (2021).

[37] W. Shiraishi, R. Yamasaki, Y. Hashimoto, S. Ko, Y. Kobayakawa, N. Isobe, T. Matsushita, and J.I. Kira, Clearance of peripheral nerve misfolded mutant protein by infiltrated macrophages correlates with motor neuron disease progression. Sci Rep 11 (2021) 16438.

[38] J. Stein, B. Walkenfort, H. Cihankaya, M. Hasenberg, V. Bader, K.F. Winklhofer, P. Röderer, J. Matschke, C. Theiss, and V. Matschke, Increased ROS-Dependent Fission of Mitochondria Causes Abnormal Morphology of the Cell Powerhouses in a Murine Model of Amyotrophic Lateral Sclerosis. Oxid Med Cell Longev 2021 (2021) 6924251.

[39] P.H. Truong, P.J. Crouch, J.B.W. Hilton, C.A. McLean, R. Cappai, and G.D. Ciccotosto, Sex-dependent effects of amyloid precursor-like protein 2 in the SOD1-G37R transgenic mouse model of MND. Cell Mol Life Sci 78 (2021) 6605-6630.

[40] J. Wang, L. Tierney, R. Mann, T. Lonsway, and C.L. Walker, Bisperoxovanadium promotes motor neuron survival and neuromuscular innervation in amyotrophic lateral sclerosis. Mol Brain 14 (2021) 155.

[41] T. Wang, D. Tomas, N.D. Perera, B. Cuic, S. Luikinga, A. Viden, S.K. Barton, C.A. McLean, A.L. Samson, A. Southon, A.I. Bush, J.M. Murphy, and B.J. Turner, Ferroptosis mediates selective motor neuron death in amyotrophic lateral sclerosis. Cell Death Differ 29 (2022) 1187-1198.

[42] J.A. Watkins, J.J.P. Alix, P.J. Shaw, and R.J. Mead, Extensive phenotypic characterisation of a human TDP-43(Q331K) transgenic mouse model of amyotrophic lateral sclerosis (ALS). Sci Rep 11 (2021) 16659.

[43] T.L. Wells, J.R. Myles, and T. Akay, C-Boutons and Their Influence on Amyotrophic Lateral Sclerosis Disease Progression. J Neurosci 41 (2021) 8088-8101.

[44] T.G. Woo, M.H. Yoon, S.M. Kang, S. Park, J.H. Cho, Y.J. Hwang, J. Ahn, H. Jang, Y.J. Shin, E.M. Jung, N.C. Ha, B.H. Kim, Y. Kwon, and B.J. Park, Novel chemical inhibitor against SOD1 misfolding and aggregation protects neuron-loss and ameliorates disease symptoms in ALS mouse model. Commun Biol 4 (2021) 1397.

[45] A.L. Wright, P.A. Della Gatta, S. Le, B.A. Berning, P. Mehta, K.R. Jacobs, H. Gul, R. San Gil, T.J. Hedl, W.R. Riddell, O. Watson, S.S. Keating, J. Venturato, R.S. Chung, J.D. Atkin, A. Lee, B. Shi, C.A. Blizzard, M. Morsch, and A.K. Walker, Riluzole does not ameliorate disease caused by cytoplasmic TDP-43 in a mouse model of amyotrophic lateral sclerosis. Eur J Neurosci 54 (2021) 6237-6255.

Validation set 1

Number of records: 31

Topic: Amyotrophic lateral sclerosis

[1] P.R. Andjus, D. Bataveljić, G. Vanhoutte, D. Mitrecic, F. Pizzolante, N. Djogo, C. Nicaise, F. Gankam Kengne, C. Gangitano, F. Michetti, A. van der Linden, R. Pochet, and G. Bacić, In vivo morphological changes in animal models of amyotrophic lateral sclerosis and Alzheimer's-like disease: MRI approach. Anatomical record (Hoboken, N.J. : 2007) 292 (2009) 1882-92.

[2] F. Angenstein, H.G. Niessen, J. Goldschmidt, S. Vielhaber, A.C. Ludolph, and H. Scheich, Age-dependent changes in MRI of motor brain stem nuclei in a mouse model of ALS. Neuroreport 15 (2004) 2271-4.

[3] D. Bataveljić, S. Stamenković, G. Bačić, and P.R. Andjus, Imaging cellular markers of neuroinflammation in the brain of the rat model of amyotrophic lateral sclerosis. Acta physiologica Hungarica 98 (2011) 27-31.

[4] P. Bigini, V. Diana, S. Barbera, E. Fumagalli, E. Micotti, L. Sitia, A. Paladini, C. Bisighini, L. De Grada, L. Coloca, L. Colombo, P. Manca, P. Bossolasco, F. Malvestiti, F. Fiordaliso, G. Forloni, M. Morbidelli, M. Salmona, D. Giardino, T. Mennini, D. Moscatelli, V. Silani, and L. Cova, Longitudinal tracking of human fetal cells labeled with super paramagnetic iron oxide nanoparticles in the brain of mice with motor neuron disease. PloS one 7 (2012) e32326.

[5] R. Bonafede, E. Turano, I. Scambi, A. Busato, P. Bontempi, F. Virla, L. Schiaffino, P. Marzola, B. Bonetti, and R. Mariotti, ASC-Exosomes Ameliorate the Disease Progression in SOD1(G93A) Murine Model Underlining Their Potential Therapeutic Use in Human ALS. International journal of molecular sciences 21 (2020).

[6] P. Bontempi, A. Busato, R. Bonafede, L. Schiaffino, I. Scambi, A. Sbarbati, R. Mariotti, and P. Marzola, MRI reveals therapeutical efficacy of stem cells: An experimental study on the SOD1(G93A) animal model. Magnetic resonance in medicine 79 (2018) 459-469.

[7] S. Bucher, K.E. Braunstein, H.G. Niessen, T. Kaulisch, M. Neumaier, T.M. Boeckers, D. Stiller, and A.C. Ludolph, Vacuolization correlates with spin-spin relaxation time in motor brainstem nuclei and behavioural tests in the transgenic G93A-SOD1 mouse model of ALS. The European journal of neuroscience 26 (2007) 1895-901.

[8] L. Canzi, V. Castellaneta, S. Navone, S. Nava, M. Dossena, I. Zucca, T. Mennini, P. Bigini, and E.A. Parati, Human skeletal muscle stem cell antiinflammatory activity ameliorates clinical outcome in amyotrophic lateral sclerosis models. Molecular medicine (Cambridge, Mass.) 18 (2012) 401-11.

[9] I. Caron, E. Micotti, A. Paladini, G. Merlino, L. Plebani, G. Forloni, M. Modo, and C. Bendotti, Comparative Magnetic Resonance Imaging and Histopathological Correlates in Two SOD1 Transgenic Mouse Models of Amyotrophic Lateral Sclerosis. PloS one 10 (2015) e0132159.

[10] J.K. Choi, A. Dedeoglu, and B.G. Jenkins, Longitudinal monitoring of motor neuron circuitry in FALS rats using in-vivo phMRI. Neuroreport 21 (2010) 157-62.

[11] G.J. Cowin, T.J. Butler, N.D. Kurniawan, C. Watson, and R.H. Wallace, Magnetic resonance microimaging of the spinal cord in the SOD1 mouse model of amyotrophic lateral sclerosis detects motor nerve root degeneration. NeuroImage 58 (2011) 69-74.

[12] M.C. Evans, P.J. Gaillard, M. de Boer, C. Appeldoorn, R. Dorland, N.R. Sibson, M.R. Turner, D.C. Anthony, and H.B. Stolp, CNS-targeted glucocorticoid reduces pathology in mouse model of amyotrophic lateral sclerosis. Acta neuropathologica communications 2 (2014) 66.

[13] M.C. Evans, S. Serres, A.A. Khrapitchev, H.B. Stolp, D.C. Anthony, K. Talbot, M.R. Turner, and N.R. Sibson, T_2-weighted MRI detects presymptomatic pathology in the SOD1 mouse model of ALS. Journal of cerebral blood flow and metabolism : official journal of the International Society of Cerebral Blood Flow and Metabolism 34 (2014) 785-93.

[14] M. Figini, A. Scotti, S. Marcuzzo, S. Bonanno, F. Padelli, V. Moreno-Manzano, J.M. García-Verdugo, P. Bernasconi, R. Mantegazza, M.G. Bruzzone, and I. Zucca, Comparison of Diffusion MRI Acquisition Protocols for the In Vivo Characterization of the Mouse Spinal Cord: Variability Analysis and Application to an Amyotrophic Lateral Sclerosis Model. PloS one 11 (2016) e0161646.

[15] J. Gao, M. Jiang, R.L. Magin, R.G. Gatto, G. Morfini, A.C. Larson, and W. Li, Multicomponent diffusion analysis reveals microstructural alterations in spinal cord of a mouse model of amyotrophic lateral sclerosis ex vivo. PloS one 15 (2020) e0231598.

[16] R.G. Gatto, M. Amin, A. Finkielsztein, C. Weissmann, T. Barrett, C. Lamoutte, O. Uchitel, R. Sumagin, T.H. Mareci, and R.L. Magin, Unveiling early cortical and subcortical neuronal degeneration in ALS mice by ultra-high field diffusion MRI. Amyotrophic lateral sclerosis & frontotemporal degeneration 20 (2019) 549-561.

[17] R.G. Gatto, W. Li, J. Gao, and R.L. Magin, In vivo diffusion MRI detects early spinal cord axonal pathology in a mouse model of amyotrophic lateral sclerosis. NMR in biomedicine 31 (2018) e3954.

[18] R.G. Gatto, W. Li, and R.L. Magin, Diffusion tensor imaging identifies presymptomatic axonal degeneration in the spinal cord of ALS mice. Brain research 1679 (2018) 45-52.

[19] R.G. Gatto, S.M. Mustafi, M.Y. Amin, T.H. Mareci, Y.C. Wu, and R.L. Magin, Neurite orientation dispersion and density imaging can detect presymptomatic axonal degeneration in the spinal cord of ALS mice. Functional neurology 33 (2018) 155-163.

[20] R.A. Grant, P.S. Sharp, A.J. Kennerley, J. Berwick, A. Grierson, T. Ramesh, and T.J. Prescott, Abnormalities in whisking behaviour are associated with lesions in brain stem nuclei in a mouse model of amyotrophic lateral sclerosis. Behavioural brain research 259 (2014) 274-83.

[21] G. Grolez, M. Kyheng, R. Lopes, C. Moreau, K. Timmerman, F. Auger, G. Kuchcinski, A. Duhamel, P. Jissendi-Tchofo, P. Besson, C. Laloux, M. Petrault, J.C. Devedjian, T. Pérez, P.F. Pradat, L. Defebvre, R. Bordet, V. Danel-Brunaud, and D. Devos, MRI of the cervical spinal cord predicts respiratory dysfunction in ALS. Scientific reports 8 (2018) 1828.

[22] Y. Jouroukhin, R. Ostritsky, Y. Assaf, G. Pelled, E. Giladi, and I. Gozes, NAP (davunetide) modifies disease progression in a mouse model of severe neurodegeneration: protection against impairments in axonal transport. Neurobiology of disease 56 (2013) 79-94.

[23] M. Majchrzak, K. Drela, A. Andrzejewska, P. Rogujski, S. Figurska, M. Fiedorowicz, P. Walczak, M. Janowski, B. Lukomska, and L. Stanaszek, SOD1/Rag2 Mice with Low Copy Number of SOD1 Gene as a New Long-Living Immunodeficient Model of ALS. Scientific reports 9 (2019) 799.

[24] S. Marcuzzo, S. Bonanno, M. Figini, A. Scotti, I. Zucca, L. Minati, N. Riva, T. Domi, A. Fossaghi, A. Quattrini, B. Galbardi, S. D'Alessandro, M.G. Bruzzone, J.M. García-Verdugo, V. Moreno-Manzano, R. Mantegazza, and P. Bernasconi, A longitudinal DTI and histological study of the spinal cord reveals early pathological alterations in G93A-SOD1 mouse model of amyotrophic lateral sclerosis. Experimental neurology 293 (2017) 43-52.

[25] S. Marcuzzo, I. Zucca, A. Mastropietro, N.K. de Rosbo, P. Cavalcante, S. Tartari, S. Bonanno, L. Preite, R. Mantegazza, and P. Bernasconi, Hind limb muscle atrophy precedes cerebral neuronal degeneration in G93A-SOD1 mouse model of amyotrophic lateral sclerosis: a longitudinal MRI study. Experimental neurology 231 (2011) 30-7.

[26] C. Moreau, V. Danel, J.C. Devedjian, G. Grolez, K. Timmerman, C. Laloux, M. Petrault, F. Gouel, A. Jonneaux, M. Dutheil, C. Lachaud, R. Lopes, G. Kuchcinski, F. Auger, M. Kyheng, A. Duhamel, T. Pérez, P.F. Pradat, H. Blasco, C. Veyrat-Durebex, P. Corcia, P. Oeckl, M. Otto, L. Dupuis, G. Garçon, L. Defebvre, Z.I. Cabantchik, J. Duce, R. Bordet, and D. Devos, Could Conservative Iron Chelation Lead to Neuroprotection in Amyotrophic Lateral Sclerosis? Antioxidants & redox signaling 29 (2018) 742-748.

[27] H.G. Niessen, F. Angenstein, K. Sander, W.S. Kunz, M. Teuchert, A.C. Ludolph, H.J. Heinze, H. Scheich, and S. Vielhaber, In vivo quantification of spinal and bulbar motor neuron degeneration in the G93A-SOD1 transgenic mouse model of ALS by T2 relaxation time and apparent diffusion coefficient. Experimental neurology 201 (2006) 293-300.

[28] C.K. Underwood, N.D. Kurniawan, T.J. Butler, G.J. Cowin, and R.H. Wallace, Non-invasive diffusion tensor imaging detects white matter degeneration in the spinal cord of a mouse model of amyotrophic lateral sclerosis. NeuroImage 55 (2011) 455-61.

[29] M.A. White, Z. Lin, E. Kim, C.M. Henstridge, E. Pena Altamira, C.K. Hunt, E. Burchill, I. Callaghan, A. Loreto, H. Brown-Wright, R. Mead, C. Simmons, D. Cash, M.P. Coleman, and J. Sreedharan, Sarm1 deletion suppresses TDP-43-linked motor neuron degeneration and cortical spine loss. Acta neuropathologica communications 7 (2019) 166.

[30] J.M. Wilson, M.S. Petrik, S.C. Grant, S.J. Blackband, J. Lai, and C.A. Shaw, Quantitative measurement of neurodegeneration in an ALS-PDC model using MR microscopy. NeuroImage 23 (2004) 336-43.

[31] D.W. Zang, Q. Yang, H.X. Wang, G. Egan, E.C. Lopes, and S.S. Cheema, Magnetic resonance imaging reveals neuronal degeneration in the brainstem of the superoxide dismutase 1 transgenic mouse model of amyotrophic lateral sclerosis. The European journal of neuroscience 20 (2004) 1745-51.

Validation set 2

Number of records: 244

Topic: Multiple sclerosis

[1] T.O.K.k. Abakumova, A. A.; Zharova, M. V.; Pozdeeva, D. A.; Gubskii, I. L.; Shepeleva, I. I.; Antonova, O. M.; Nukolova, N. V.; Kekelidze, Z. I.; Chekhonin, V. P., Cuprizone Model as a Tool for Preclinical Studies of the Efficacy of Multiple Sclerosis Diagnosis and Therapy. Bulletin of Experimental Biology and Medicine 159 (2015) 111-115.

[2] P.K. Abramowski, S.; Ernst, T.; Lange, C.; Ittrich, H.; Schweizer, M.; Zander, A. R.; Martin, R.; Fehse, B., Mesenchymal Stromal/Stem Cells Do Not Ameliorate Experimental Autoimmune Encephalomyelitis and Are Not Detectable in the Central Nervous System of Transplanted Mice. Stem Cells Dev 25 (2016) 1134-48.

[3] P.K. Acs, M.; Norkute, A.; Johann, S.; Clarner, T.; Braun, A.; Berente, Z.; Komoly, S.; Beyer, C., 17beta-estradiol and progesterone prevent cuprizone provoked demyelination of corpus callosum in male mice. Glia 57 (2009) 807-14.

[4] S.M. Adler, J.; Williams, D. S.; Verbalis, J. G., Positive association between blood brain barrier disruption and osmotically-induced demyelination. Multiple Sclerosis 6 (2000) 24-31.

[5] S.V. Adler, J. G.; Williams, D., Effect of rapid correction of hyponatremia on the blood-brain barrier of rats. Brain Research 679 (1995) 135-43.

[6] S.V. Adler, J. G.; Meyers, S.; Simplaceanu, E.; Williams, D. S., Changes in cerebral blood flow and distribution associated with acute increases in plasma sodium and osmolality of chronic hyponatremic rats. Experimental Neurology 163 (2000) 63-71.

[7] M.J. Aggarwal, M. V.; Calabresi, P. A.; Mori, S.; Zhang, J., Probing mouse brain microstructure using oscillating gradient diffusion MRI. Magnetic Resonance in Medicine 67 (2012) 98-109.

[8] R.S. Aharoni, E.; Blumenfeld-Katzir, T.; Eilam, R.; Sela, M.; Assaf, Y.; Arnon, R., Magnetic resonance imaging characterization of different experimental autoimmune encephalomyelitis models and the therapeutic effect of glatiramer acetate. Experimental Neurology 240 (2013) 130-44.

[9] E.T.L. Ahrens, D. H.; Readhead, C.; Brosnan, C. F.; Fraser, S. E.; Jacobs, R. E., MR microscopy of transgenic mice that spontaneously acquire experimental allergic encephalomyelitis. Magnetic Resonance in Medicine 40 (1998) 119-32.

[10] E.M. Aizman, A.; Chapman, J.; Assaf, Y.; Kloog, Y., The combined treatment of Copaxone and Salirasib attenuates experimental autoimmune encephalomyelitis (EAE) in mice. Journal of neuroimmunology 229 (2010) 192-203.

[11] A.H.W. All, P.; Agrawal, G.; Gorelik, M.; Lee, C.; Thakor, N. V.; Bulte, J. W.; Kerr, D. A., Effect of MOG sensitization on somatosensory evoked potential in Lewis rats. Journal of the Neurological Sciences 284 (2009) 81-9.

[12] S.A.S.-Q. Anderson, J.; Jordan, E. K.; Arbab, A. S.; Martin, R.; McFarland, H.; Frank, J. A., Magnetic resonance imaging of labeled T-cells in a mouse model of multiple sclerosis. Annals of Neurology 55 (2004) 654-9.

[13] D.C.S. Anthony, N. R.; McAteer, M. A.; Davis, B.; Choudhury, R. P., Detection of brain pathology by magnetic resonance imaging of iron oxide micro-particles. Methods Mol Biol 686 (2011) 213-27.

[14] D.C.S. Anthony, N. R.; Losey, P.; Meier, D. P.; Leppert, D., Investigation of immune and CNS-mediated effects of fingolimod in the focal delayed-type hypersensitivity multiple sclerosis model. Neuropharmacology 79 (2014) 534-41.

[15] M.K.B. Axthelm, D. N.; Marracci, G. H.; Su, W.; Mullaney, E. T.; Manoharan, M.; Kohama, S. G.; Pollaro, J.; Witkowski, E.; Wang, P.; Rooney, W. D.; Sherman, L. S.; Wong, S. W., Japanese macaque encephalomyelitis: a spontaneous multiple sclerosis-like disease in a nonhuman primate. Annals of Neurology 70 (2011) 362-73.

[16] A.H.K. Badawi, P.; Wang, W. T.; Choi, I. Y.; Lee, P.; Vines, C. M.; Siahaan, T. J., Suppression of EAE and prevention of blood-brain barrier breakdown after vaccination with novel bifunctional peptide inhibitor. Neuropharmacology 62 (2012) 1874-81.

[17] K.A. Baeten, P.; Hendriks, J.; Theunissen, E.; Gelan, J.; Hellings, N.; Stinissen, P., Tracking of myelin-reactive T cells in experimental autoimmune encephalomyelitis (EAE) animals using small particles of iron oxide and MRI. NMR in biomedicine 23 (2010) 601-9.

[18] K.H. Baeten, J. J.; Hellings, N.; Theunissen, E.; Vanderlocht, J.; Ryck, L. D.; Gelan, J.; Stinissen, P.; Adriaensens, P., Visualisation of the kinetics of macrophage infiltration during experimental autoimmune encephalomyelitis by magnetic resonance imaging. Journal of neuroimmunology 195 (2008) 1-6.

[19] J.C.L. Bell, Q.; Gan, Y.; Liu, Q.; Liu, Y.; Shi, F. D.; Turner, G. H., Visualization of inflammation and demyelination in 2D2 transgenic mice with rodent MRI. Journal of neuroimmunology 264 (2013) 35-40.

[20] T.v.H. Ben-Hur, R. B.; Einstein, O.; Aharonowiz, M.; Xue, R.; Frost, E. E.; Mori, S.; Reubinoff, B. E.; Bulte, J. W., Serial in vivo MR tracking of magnetically labeled neural spheres transplanted in chronic EAE mice. Magnetic Resonance in Medicine 57 (2007) 164-71.

[21] M.L. Bendszus, G.; Jestaedt, L.; Misselwitz, B.; Solymosi, L.; Toyka, K.; Stoll, G., Gadofluorine M enhancement allows more sensitive detection of inflammatory CNS lesions than T2-w imaging: a quantitative MRI study. Brain 131 (2008) 2341-52.

[22] E.V. Beraud, A.; Regaya, I.; Confort-Gouny, S.; Siaud, P.; Ibarrola, D.; Le Fur, Y.; Barbaria, J.; Pellissier, J. F.; Sabatier, J. M.; Medina, I.; Cozzone, P. J., Block of neural Kv1.1 potassium channels for neuroinflammatory disease therapy. Annals of Neurology 60 (2006) 586-96.

[23] C.H. Berger, P.; Kindler-Baumann, D.; Rudin, M.; Rausch, M., Analysis of lesion development during acute inflammation and remission in a rat model of experimental autoimmune encephalomyelitis by visualization of macrophage infiltration, demyelination and blood-brain barrier damage. NMR in biomedicine 19 (2006) 101-7.

[24] I.E.M. Biton, A.; Kidron, D.; Assaf, Y.; Cohen, Y., Improved detectability of experimental allergic encephalomyelitis in excised swine spinal cords by high b-value q-space DWI. Experimental Neurology 195 (2005) 437-46.

[25] S.M. Bittner, S. G.; Göbel, K.; Melzer, N.; Herrmann, A. M.; Simon, O. J.; Weishaupt, A.; Budde, T.; Bayliss, D. A.; Bendszus, M.; Wiendl, H., TASK1 modulates inflammation and neurodegeneration in autoimmune inflammation of the central nervous system. Brain 132 (2009) 2501-2516.

[26] T.C.M. Blair, M.; Rawlings-Rhea, S. D.; Tagge, I.; Kohama, S. G.; Hollister-Smith, J.; Ferguson, B.; Woltjer, R. L.; Frederick, M. C.; Pollaro, J.; Rooney, W. D.; Sherman, L. S.; Bourdette, D. N.; Wong, S. W., Immunopathology of Japanese macaque encephalomyelitis is similar to multiple sclerosis. Journal of neuroimmunology 291 (2016) 1-10.

[27] E.L.B. Blezer, J.; Brok, H. P.; Nicolay, K.; t Hart, B. A., Quantitative MRI-pathology correlations of brain white matter lesions developing in a non-human primate model of multiple sclerosis. NMR in biomedicine 20 (2007) 90-103.

[28] E.L.D. Blezer, L. H.; Kooij, G.; Drexhage, J.; van der Pol, S. M.; Reijerkerk, A.; Dijkhuizen, R. M.; de Vries, H. E., In vivo MR imaging of intercellular adhesion molecule-1 expression in an animal model of multiple sclerosis. Contrast Media Mol Imaging 10 (2015) 111-21.

[29] S.E. Boretius, A.; Dallenga, T.; Wrzos, C.; Tammer, R.; Bruck, W.; Nessler, S.; Frahm, J.; Stadelmann, C., Assessment of lesion pathology in a new animal model of MS by multiparametric MRI and DTI. NeuroImage 59 (2012) 2678-88.

[30] S.G. Boretius, I.; Demmer, I.; Bahr, M.; Diem, R.; Michaelis, T.; Frahm, J., MRI of optic neuritis in a rat model. NeuroImage 41 (2008) 323-34.

[31] S.S. Boretius, B.; Watanabe, T.; Merkler, D.; Tammer, R.; Czeh, B.; Michaelis, T.; Frahm, J.; Fuchs, E., Monitoring of EAE onset and progression in the common marmoset monkey by sequential high-resolution 3D MRI. NMR in biomedicine 19 (2006) 41-9.

[32] B.D. Brochet, V., Pathological correlates of magnetization transfer imaging abnormalities in animal models and humans with multiple sclerosis. Neurology 53 (1999) S12-7.

[33] B.D. Brochet, M. S.; Touil, T.; Anne, O.; Caille, J. M.; Dousset, V.; Petry, K. G., Early macrophage MRI of inflammatory lesions predicts lesion severity and disease development in relapsing EAE. NeuroImage 32 (2006) 266-74.

[34] H.P.M.V.M. Brok, M.; Blezer, E.; Schantz, A.; Peritt, D.; Treacy, G.; Laman, J. D.; Bauer, J.; T Hart, B. A., Prevention of experimental autoimmune encephalomyelitis in common marmosets using an anti-IL-12p40 monoclonal antibody. Journal of Immunology 169 (2002) 6554-6563.

[35] K.A.A. Broom, D. C.; Blamire, A. M.; Waters, S.; Styles, P.; Perry, V. H.; Sibson, N. R., MRI reveals that early changes in cerebral blood volume precede blood-brain barrier breakdown and overt pathology in MS-like lesions in rat brain. J Cereb Blood Flow Metab 25 (2005) 204-16.

[36] M.D.K. Budde, J. H.; Liang, H. F.; Russell, J. H.; Cross, A. H.; Song, S. K., Axonal injury detected by in vivo diffusion tensor imaging correlates with neurological disability in a mouse model of multiple sclerosis. NMR in biomedicine 21 (2008) 589-97.

[37] M.D.X. Budde, M.; Cross, A. H.; Song, S. K., Axial diffusivity is the primary correlate of axonal injury in the experimental autoimmune encephalomyelitis spinal cord: a quantitative pixelwise analysis. Journal of Neuroscience 29 (2009) 2805-13.

[38] J.W.B.-H. Bulte, T.; Miller, B. R.; Mizrachi-Kol, R.; Einstein, O.; Reinhartz, E.; Zywicke, H. A.; Douglas, T.; Frank, J. A., MR microscopy of magnetically labeled neurospheres transplanted into the Lewis EAE rat brain. Magnetic Resonance in Medicine 50 (2003) 201-5.

[39] A.F. Carambia, B.; Schwinge, D.; Bruns, O. T.; Salmen, S. C.; Ittrich, H.; Reimer, R.; Heine, M.; Huber, S.; Waurisch, C.; Eychmüller, A.; Wraith, D. C.; Korn, T.; Nielsen, P.; Weller, H.; Schramm, C.; Lüth, S.; Lohse, A. W.; Heeren, J.; Herkel, J., Nanoparticle-based autoantigen delivery to Treg-inducing liver sinusoidal endothelial cells enables control of autoimmunity in mice. Journal of Hepatology 62 (2015) 1349-1356.

[40] H.S.W. Cate, Q. Z.; Kemper, D.; Merlo, D.; Wang, H. X.; Fang, K.; Egan, G. F.; Kilpatrick, T. J., Influence of methylprednisolone on magnetic resonance and histological measures during cuprizone-induced demyelination. Neuroscience Letters 483 (2010) 47-52.

[41] P.U. Chandran, J.; Markosyan, S.; Lisowski, A.; Buck, W.; Chin, C. L.; Fox, G.; Luo, F.; Day, M., Magnetic resonance imaging and histological evidence for the blockade of cuprizone-induced demyelination in C57BL/6 mice. Neuroscience 202 (2012) 446-53.

[42] C.C.Z. Chen, A.; Hsu, Y. H.; Chen, H. W.; Yang, L. C.; Chang, C., Neuroaxonal ion dyshomeostasis of the normal-appearing corpus callosum in experimental autoimmune encephalomyelitis. Experimental Neurology 210 (2008) 322-30.

[43] J.W.B. Chen, M. O.; Aikawa, E.; Chiang, G.; Weissleder, R., Myeloperoxidase-targeted imaging of active inflammatory lesions in murine experimental autoimmune encephalomyelitis. Brain 131 (2008) 1123-33.

[44] X.H. Chen, X.; Zou, Y.; Pi, R.; Liu, M.; Wang, T.; Zheng, X.; Liu, M.; Lin, M.; Liu, P.; Tao, L., Combined treatment with minocycline and prednisone attenuates experimental autoimmune encephalomyelitis in C57 BL/6 mice.[Erratum appears in J Neuroimmunol. 2009 Oct 30;215(1-2):130]. Journal of neuroimmunology 210 (2009) 22-9.

[45] X.P. Chen, R.; Zou, Y.; Liu, M.; Ma, X.; Jiang, Y.; Mao, X.; Hu, X., Attenuation of experimental autoimmune encephalomyelitis in C57 BL/6 mice by osthole, a natural coumarin. Eur J Pharmacol 629 (2010) 40-6.

[46] Z.C. Chen, J. T.; Johnson, M.; Gossman, Z. C.; Hendrickson, M.; Sakaie, K.; Martinez-Rubio, C.; Gale, J. T.; Trapp, B. D., Cuprizone does not induce CNS demyelination in nonhuman primates. Ann 2 (2015) 208-13.

[47] C.L.P. Chin, M.; Bousquet, P. F.; Schwartz, A. J.; O'Connor, E. M.; Nelson, C. M.; Hradil, V. P.; Cox, B. F.; McRae, B. L.; Fox, G. B., Distinct spatiotemporal pattern of CNS lesions revealed by USPIO-enhanced MRI in MOG-induced EAE rats implicates the involvement of spino-olivocerebellar pathways. Journal of neuroimmunology 211 (2009) 49-55.

[48] N.C. Collongues, J. B.; Blanc, F.; Steibel, J.; Lam, C. D.; Shabbir, A.; Trifilieff, E.; Honnorat, J.; Pham-Dinh, D.; Ghandour, M. S.; de Seze, J., The Brown Norway opticospinal model of demyelination: does

it mimic multiple sclerosis or neuromyelitis optica? International Journal of Developmental Neuroscience 30 (2012) 487-97.

[49] L.L.F. Cook, P. J.; Mitchell, J. R.; Karlik, S. J., In vivo 4.0-T magnetic resonance investigation of spinal cord inflammation, demyelination, and axonal damage in chronic-progressive experimental allergic encephalomyelitis. Journal of Magnetic Resonance Imaging 20 (2004) 563-71.

[50] L.L.F. Cook, P. J.; Karlik, S. J., Pathology-guided MR analysis of acute and chronic experimental allergic encephalomyelitis spinal cord lesions at 1.5T. Journal of Magnetic Resonance Imaging 22 (2005) 180-8.

[51] L.C. Cruz-Orengo, Y. J.; Kim, J. H.; Dorsey, D.; Song, S. K.; Klein, R. S., CXCR7 antagonism prevents axonal injury during experimental autoimmune encephalomyelitis as revealed by in vivo axial diffusivity. Journal of Neuroinflammation (2011) 170.

[52] A.K.R. de Santana Nunes, C.; de Oliveira, W. H.; Thomé, R.; Verinaud, L.; Tovar-Moll, F.; Peixoto, C. A., Phosphodiesterase-5 inhibition promotes remyelination by MCP-1/CCR-2 and MMP-9 regulation in a cuprizone-induced demyelination model. Experimental Neurology 275 (2016) 143-153.

[53] C.A.Z. DeBoy, J.; Dike, S.; Shats, I.; Jones, M.; Reich, D. S.; Mori, S.; Nguyen, T.; Rothstein, B.; Miller, R. H.; Griffin, J. T.; Kerr, D. A.; Calabresi, P. A., High resolution diffusion tensor imaging of axonal damage in focal inflammatory and demyelinating lesions in rat spinal cord. Brain 130 (2007) 2199-210.

[54] M.N.J. Degaonkar, R.; Jagannathan, N. R., Sequential diffusion-weighted magnetic resonance imaging study of lysophosphatidyl choline-induced experimental demyelinating lesion: an animal model of multiple sclerosis. Journal of Magnetic Resonance Imaging 16 (2002) 153-9.

[55] M.N.R. Degaonkar, P.; Jayasundar, R.; Jagannathan, N. R., Determination of relaxation characteristics during preacute stage of lysophosphatidyl choline-induced demyelinating lesion in rat brain: an animal model of multiple sclerosis. Magnetic Resonance Imaging 23 (2005) 69-73.

[56] M.S.T. Deloire, T.; Brochet, B.; Dousset, V.; Caille, J. M.; Petry, K. G., Macrophage brain infiltration in experimental autoimmune encephalomyelitis is not completely compromised by suppressed T-cell invasion: in vivo magnetic resonance imaging illustration in effective anti-VLA-4 antibody treatment. Multiple Sclerosis 10 (2004) 540-8.

[57] M.S.B. Deloire-Grassin, B.; Quesson, B.; Delalande, C.; Dousset, V.; Canioni, P.; Petry, K. G., In vivo evaluation of remyelination in rat brain by magnetization transfer imaging. Journal of the Neurological Sciences 178 (2000) 10-6.

[58] R.A.D. Desai, A. L.; Tachrount, M.; Kasti, M.; Laulund, F.; Golay, X.; Smith, K. J., Cause and prevention of demyelination in a model multiple sclerosis lesion. Annals of Neurology 79 (2016) 591-604.

[59] R.D. Diem, I.; Boretius, S.; Merkler, D.; Schmelting, B.; Williams, S. K.; Sattler, M. B.; Bahr, M.; Michaelis, T.; Frahm, J.; Bruck, W.; Fuchs, E., Autoimmune optic neuritis in the common marmoset monkey: comparison of visual evoked potentials with MRI and histopathology. Investigative Ophthalmology & Visual Science 49 (2008) 3707-14.

[60] R.L. Dommisse, D.; Spanoghe, M.; Van Audekerke, J.; Van der Linden, A.; Gravenmade, H.; Van de Vyver, F., Optimalization of NMR micro imaging (1.9T, 4.7T, 7.4T) of the central nervous system of an animal model for multiple sclerosis, Proceedings of the 13th Annual International Conference of the IEEE Engineering in Medicine and Biology Society, Publ by IEEE, Piscataway, NJ, United States; Orlando, FL, USA, 1991, pp. 79-80.

[61] V.B. Dousset, B.; Vital, A.; Gross, C.; Benazzouz, A.; Boullerne, A.; Bidabe, A. M.; Gin, A. M.; Caille, J. M., Lysolecithin-induced demyelination in primates: preliminary in vivo study with MR and magnetization transfer. AJNR Am J Neuroradiol 16 (1995) 225-31.

[62] V.D. Dousset, C.; Ballarino, L.; Quesson, B.; Seilhan, D.; Coussemacq, M.; Thiaudiere, E.; Brochet, B.; Canioni, P.; Caille, J. M., In vivo macrophage activity imaging in the central nervous system detected by magnetic resonance. Magnetic Resonance in Medicine 41 (1999) 329-33.

[63] H.J.M. Duckers, H. J.; Verhaagen, J.; Nicolay, K.; Gispen, W. H., Longitudinal in vivo magnetic resonance imaging studies in experimental allergic encephalomyelitis: effect of a neurotrophic treatment on cortical lesion development. Neuroscience 77 (1997) 1163-73.

[64] R.D.v.d.P. Engberink, S. M.; Walczak, P.; van der Toorn, A.; Viergever, M. A.; Dijkstra, C. D.; Bulte, J. W.; de Vries, H. E.; Blezer, E. L., Magnetic resonance imaging of monocytes labeled with ultrasmall superparamagnetic particles of iron oxide using magnetoelectroporation in an animal model of multiple sclerosis. Molecular Imaging 9 (2010) 268-77.

[65] G.D.A. Esposito, L.; Bartoli, A.; Chaabane, L.; Terreno, E., Evaluation of the co-registration capabilities of a MRI/PET compatible bed in an Experimental autoimmune encephalomyelitis (EAE) model. Nucl. Instrum. Methods Phys. Res. Sect. A-Accel. Spectrom. Dect. Assoc. Equip. 702 (2013) 108-110.

[66] M.F.G. Falangola, D. N.; Tabesh, A.; Hui, E. S.; Nie, X.; Jensen, J. H.; Gerum, S. V.; Hu, C.; Lafrancois, J.; Collins, H. R.; Helpern, J. A., Histological correlation of diffusional kurtosis and white matter modeling metrics in cuprizone-induced corpus callosum demyelination. NMR in biomedicine 27 (2014) 948-957.

[67] M.H. Fang, D.; Zhang, F.; Hu, Z.; Yang, J.; Jiang, H.; Han, S., Antineuroinflammatory and neurotrophic effects of CNTF and C16 peptide in an acute experimental autoimmune encephalomyelitis rat models. Frontiers in Neuroanatomy 7 (2013).

[68] S.B. Fjær, L.; Lundervold, A.; Myhr, K. M.; Pavlin, T.; Torkildsen, Ø; Wergeland, S., Deep gray matter demyelination detected by magnetization transfer ratio in the cuprizone model. PLoS ONE 8 (2013).

[69] S.B. Fjaer, L.; Myhr, K. M.; Torkildsen, O.; Wergeland, S., Magnetization transfer ratio does not correlate to myelin content in the brain in the MOG-EAE mouse model. Neurochemistry International 83-84 (2015) 28-40.

[70] S.B. Floris, E. L.; Schreibelt, G.; Dopp, E.; van der Pol, S. M.; Schadee-Eestermans, I. L.; Nicolay, K.; Dijkstra, C. D.; de Vries, H. E., Blood-brain barrier permeability and monocyte infiltration in experimental allergic encephalomyelitis: a quantitative MRI study. Brain 127 (2004) 616-27.

[71] C.C.C. Ford, T. L.; Karp, J.; Herndon, R. M., Magnetic resonance imaging of experimental demyelinating lesions. Magnetic Resonance in Medicine 14 (1990) 461-81.

[72] I.B. Gadjanski, S.; Williams, S. K.; Lingor, P.; Knoferle, J.; Sattler, M. B.; Fairless, R.; Hochmeister, S.; Suhs, K. W.; Michaelis, T.; Frahm, J.; Storch, M. K.; Bahr, M.; Diem, R., Role of n-type voltage-dependent calcium channels in autoimmune optic neuritis. Annals of Neurology 66 (2009) 81-93.

[73] M.I.M. Gaitan, P.; Wohler, J.; Leibovitch, E.; Sati, P.; Calandri, I. L.; Merkle, H.; Massacesi, L.; Silva, A. C.; Jacobson, S.; Reich, D. S., Perivenular brain lesions in a primate multiple sclerosis model at 7-tesla magnetic resonance imaging. Multiple Sclerosis 20 (2014) 64-71.

[74] P.J.R. Gareau, B. K.; Karlik, S. J.; Mitchell, J. R., Magnetization transfer and multicomponent T2 relaxation measurements with histopathologic correlation in an experimental model of MS. Journal of Magnetic Resonance Imaging 11 (2000) 586-95.

[75] P.J.W. Gareau, A. C.; Cofer, G. P.; Johnson, G. A., Imaging inflammation: direct visualization of perivascular cuffing in EAE by magnetic resonance microscopy. Journal of Magnetic Resonance Imaging 16 (2002) 28-36.

[76] F.C. Gilli, X.; Pachner, A. R.; Gimi, B., High-Resolution Diffusion Tensor Spinal Cord MRI Measures as Biomarkers of Disability Progression in a Rodent Model of Progressive Multiple Sclerosis. PLoS ONE 11 (2016) e0160071.

[77] K.W. Gobel, J. H.; Herrmann, A. M.; Wachsmuth, L.; Pankratz, S.; Bittner, S.; Budde, T.; Kleinschnitz, C.; Faber, C.; Wiendl, H.; Meuth, S. G., 4-Aminopyridine ameliorates mobility but not disease course in an animal model of multiple sclerosis. Experimental Neurology 248 (2013) 62-71.

[78] R.I.L. Grossman, R. P.; Macchi, P. J.; Joseph, P. M., MR of acute experimental allergic encephalomyelitis. AJNR Am J Neuroradiol 8 (1987) 1045-8.

[79] C.L.B. Guglielmetti, D.; Santermans, E.; Salas-Perdomo, A.; Daans, J.; De Vocht, N.; Shah, D.; Hoornaert, C.; Praet, J.; Peerlings, J.; Kara, F.; Bigot, C.; Mai, Z.; Goossens, H.; Hens, N.; Hendrix, S.; Verhoye, M.; Planas, A. M.; Berneman, Z.; van der Linden, A.; Ponsaerts, P., Interleukin-13 immune gene therapy prevents CNS inflammation and demyelination via alternative activation of microglia and macrophages. Glia 64 (2016) 2181-2200.

[80] C.V. Guglielmetti, J.; Roelant, E.; Mai, Z.; Daans, J.; Van Audekerke, J.; Naeyaert, M.; Vanhoutte, G.; Delgado, Y. Palacios R.; Praet, J.; Fieremans, E.; Ponsaerts, P.; Sijbers, J.; Van der Linden, A.; Verhoye, M., Diffusion kurtosis imaging probes cortical alterations and white matter pathology following cuprizone induced demyelination and spontaneous remyelination. NeuroImage 125 (2016) 363-77.

[81] J.M. Guy, S.; Fitzsimmons, J.; Beck, B.; Rao, N. A., Disruption of the blood-brain barrier in experimental optic neuritis: immunocytochemical co-localization of H2O2 and extravasated serum albumin. Investigative Ophthalmology & Visual Science 35 (1994) 1114-23.

[82] J.R.S. Guy, P.; Leibovitch, E.; Jacobson, S.; Silva, A. C.; Reich, D. S., Custom fit 3D-printed brain holders for comparison of histology with MRI in marmosets. Journal of Neuroscience Methods 257 (2016) 55-63.

[83] K.G.H. Haanstra, S. O.; Estêvão, D. M. L.; Blezer, E. L. A.; Bauer, J.; Yang, L. L.; Wyant, T.; Csizmadia, V.; T Hart, B. A.; Fedykx, E. R., Antagonizing the α4β1 integrin, but not α4β7, inhibits leukocytic infiltration of the central nervous system in rhesus monkey experimental autoimmune encephalomyelitis. Journal of Immunology 190 (2013) 1961-1973.

[84] J.S. Hao, A. R.; Turner, G. H.; Wu, J.; Whiteaker, P.; Lukas, R. J.; Shi, F. D., Attenuation of CNS inflammatory responses by nicotine involves alpha7 and non-alpha7 nicotinic receptors. Experimental Neurology 227 (2011) 110-9.

[85] L.A.S. Harsan, J.; Zaremba, A.; Agin, A.; Sapin, R.; Poulet, P.; Guignard, B.; Parizel, N.; Grucker, D.; Boehm, N.; Miller, R. H.; Ghandour, M. S., Recovery from chronic demyelination by thyroid hormone therapy: myelinogenesis induction and assessment by diffusion tensor magnetic resonance imaging. Journal of Neuroscience 28 (2008) 14189-201.

[86] B.A.B. Hart, J.; Muller, H. J.; Melchers, B.; Nicolay, K.; Brok, H.; Bontrop, R. E.; Lassmann, H.; Massacesi, L., Histopathological characterization of magnetic resonance imaging-detectable brain

white matter lesions in a primate model of multiple sclerosis: a correlative study in the experimental autoimmune encephalomyelitis model in common marmosets (Callithrix jacchus). American Journal of Pathology 153 (1998) 649-63.

[87] C.P.M. Hawkins, P. M.; MacKenzie, F.; Kesselring, J.; Tofts, P. S.; du Boulay, E. P.; Landon, D. N.; McDonald, W. I., Duration and selectivity of blood-brain barrier breakdown in chronic relapsing experimental allergic encephalomyelitis studied by gadolinium-DTPA and protein markers. Brain 113 (1990) 365-78.

[88] C.P.M. Hawkins, P. M.; Landon, D. N.; McDonald, W. I., Metabolically dependent blood-brain barrier breakdown in chronic relapsing experimental allergic encephalomyelitis. Acta Neuropathologica 83 (1992) 630-5.

[89] S.N. Heckl, T.; Herrmann, M.; Gartner, S.; Klose, U.; Schick, F.; Weissert, R.; Kuker, W., Experimental autoimmune encephalomyelitis (EAE): lesion visualization on a 3 Tesla clinical whole-body system after intraperitoneal contrast injection. ROFO Fortschr Geb Rontgenstr Nuklearmed 176 (2004) 1549-54.

[90] A.C.R. Heide, T. L.; Alvord, E. C., Jr.; Peterson, J.; Rose, L. M., Diffusion imaging of experimental allergic encephalomyelitis. Magnetic Resonance in Medicine 29 (1993) 478-84.

[91] G.G.-R. Helms, E.; Schlumbohm, C.; Konig, J.; Dechent, P.; Fuchs, E.; Wilke, M., Structural and quantitative neuroimaging of the common marmoset monkey using a clinical MRI system. Journal of Neuroscience Methods 215 (2013) 121-31.

[92] S.L.P. Herrera, V. L.; Whittaker, H.; Smith, B. C.; Kim, A.; Schellenberg, A. E.; Thiessen, J. D.; Buist, R.; Del Bigio, M. R.; Martin, M., Damage to the optic chiasm in myelin oligodendrocyte glycoprotein-experimental autoimmune encephalomyelitis mice. Magnetic Resonance Insights 7 (2014) 23-31.

[93] D.B.W. Hoffmann, S. K.; Bojcevski, J.; Muller, A.; Stadelmann, C.; Naidoo, V.; Bahr, B. A.; Diem, R.; Fairless, R., Calcium influx and calpain activation mediate preclinical retinal neurodegeneration in autoimmune optic neuritis. J Neuropathol Exp Neurol 72 (2013) 745-57.

[94] M.B. Hunger, E.; Zhong, K.; Angenstein, F., Visualization of acute focal lesions in rats with experimental autoimmune encephalomyelitis by magnetic nanoparticles, comparing different MRI sequences including phase imaging. Journal of Magnetic Resonance Imaging 39 (2014) 1126-35.

[95] S.A.H. Jagessar, N.; Bauer, J.; Blezer, E. L. A.; Laman, J. D.; Hellings, N.; T Hart, B. A., B-Cell depletion abrogates T cell-mediated demyelination in an antibody-nondependent common marmoset experimental autoimmune encephalomyelitis model. Journal of Neuropathology and Experimental Neurology 71 (2012) 716-728.

[96] S.A.K. Jagessar, Y. S.; Heijmans, N.; van Driel, N.; van Straalen, L.; Bajramovic, J. J.; Brok, H. P.; Blezer, E. L.; Bauer, J.; Laman, J. D.; t Hart, B. A., Induction of progressive demyelinating autoimmune encephalomyelitis in common marmoset monkeys using MOG34-56 peptide in incomplete freund adjuvant. J Neuropathol Exp Neurol 69 (2010) 372-85.

[97] S.A.S. Jagessar, P. A.; Blezer, E.; Delarasse, C.; Pham-Dinh, D.; Laman, J. D.; Bauer, J.; Amor, S.; t Hart, B., Autoimmunity against myelin oligodendrocyte glycoprotein is dispensable for the initiation although essential for the progression of chronic encephalomyelitis in common marmosets. J Neuropathol Exp Neurol 67 (2008) 326-40.

[98] R.P. Jaini, D. C.; Flask, C. A.; Macklin, W. B.; Tuohy, V. K., Myelin antigen load influences antigen presentation and severity of central nervous system autoimmunity. Journal of neuroimmunology 259 (2013) 37-46.

[99] V.A.Z. Janve, Z.; Yao, S. Y.; Li, K.; Zhang, F. L.; Wilson, K. J.; Ou, X.; Does, M. D.; Subramaniam, S.; Gochberg, D. F., The radial diffusivity and magnetization transfer pool size ratio are sensitive markers for demyelination in a rat model of type III multiple sclerosis (MS) lesions. NeuroImage 74 (2013) 298-305.

[100] I.O.Z. Jelescu, M.; Winters, K. V.; Veraart, J.; Rajaratnam, A.; Kim, N. S.; Babb, J. S.; Shepherd, T. M.; Novikov, D. S.; Kim, S. G.; Fieremans, E., In vivo quantification of demyelination and recovery using compartment-specific diffusion MRI metrics validated by electron microscopy. NeuroImage 132 (2016) 104-14.

[101] Y.Z. Jiang, Y.; Chen, S.; Zhu, C.; Wu, A.; Liu, Y.; Ma, L.; Zhu, D.; Ma, X.; Liu, M.; Kang, Z.; Pi, R.; Peng, F.; Wang, Q.; Chen, X., The anti-inflammatory effect of donepezil on experimental autoimmune encephalomyelitis in C57 BL/6 mice. Neuropharmacology 73 (2013) 415-24.

[102] H.L.W. Johnson, R. C.; Jin, F.; Manhart, W. A.; LaFrance, S. J.; Pirko, I.; Johnson, A. J., Perforin competent CD8 T cells are sufficient to cause immune-mediated blood-brain barrier disruption. PLoS ONE 9 (2014) e111401.

[103] E.K.M. Jordan, H. I.; Lewis, B. K.; Tresser, N.; Gates, M. A.; Johnson, M.; Lenardo, M.; Matis, L. A.; McFarland, H. F.; Frank, J. A., Serial MR imaging of experimental autoimmune encephalomyelitis induced by human white matter or by chimeric myelin-basic and proteolipid protein in the common marmoset. AJNR Am J Neuroradiol 20 (1999) 965-76.

[104] Y.S.B. Kap, J.; Driel, Nv; Bleeker, W. K.; Parren, P. W.; Kooi, E. J.; Geurts, J. J.; Laman, J. D.; Craigen, J. L.; Blezer, E.; t Hart, B. A., B-cell depletion attenuates white and gray matter pathology in marmoset experimental autoimmune encephalomyelitis. J Neuropathol Exp Neurol 70 (2011) 992-1005.

[105] Y.S.V.D. Kap, N.; Blezer, E.; Parren, P. W. H. I.; Bleeker, W. K.; Laman, J. D.; Craigen, J. L.; T Hart, B. A., Late B cell depletion with a human anti-human CD20 IgG1κ monoclonal antibody halts the development of experimental autoimmune encephalomyelitis in marmosets. Journal of Immunology 185 (2010) 3990-4003.

[106] S.J.G. Karlik, J. J.; Wong, C.; Vandervoort, M. K.; Noseworthy, J. H., NMR studies in experimental allergic encephalomyelitis: factors which contribute to T1 and T2 values. Magnetic Resonance in Medicine 14 (1990) 1-11.

[107] S.J.G. Karlik, E. A.; Lee, D.; Noseworthy, J. H., Gadolinium enhancement in acute and chronic-progressive experimental allergic encephalomyelitis in the guinea pig. Magnetic Resonance in Medicine 30 (1993) 326-31.

[108] S.J.M. Karlik, D.; St Louis, J.; Strejan, G., Correlation between MRI and clinico-pathological manifestations in Lewis rats protected from experimental allergic encephalomyelitis by acylated synthetic peptide of myelin basic protein. Magnetic Resonance Imaging 17 (1999) 731-7.

[109] S.J.S. Karlik, G.; Gilbert, J. J.; Noseworthy, J. H., NMR studies in experimental allergic encephalomyelitis (EAE): normalization of T1 and T2 with parenchymal cellular infiltration. Neurology 36 (1986) 1112-4.

[110] S.J.K. Kent, S. J.; Rice, G. P.; Horner, H. C., A monoclonal antibody to alpha 4-integrin reverses the MR-detectable signs of experimental allergic encephalomyelitis in the guinea pig. Journal of Magnetic Resonance Imaging 5 (1995) 535-40.

[111] M.G. Khodanovich, V.; Pan, E.; Akulov, A.; Krutenkova, E.; Trusov, V.; Yarnykh, V.; Iop,, MRI study of the cuprizone-induced mouse model of multiple sclerosis: demyelination is not found after co-

treatment with polyprenols (long-chain isoprenoid alcohols), 2nd International Conference and Young Scientist School ''Magnetic Resonance Imaging in Biomedical Research'', Iop Publishing Ltd, Bristol, 2016.

[112] H.W. Kim, P.; Kerr, C.; Galpoththawela, C.; Gilad, A. A.; Muja, N.; Bulte, J. W., Immunomodulation by transplanted human embryonic stem cell-derived oligodendroglial progenitors in experimental autoimmune encephalomyelitis. Stem Cells 30 (2012) 2820-9.

[113] H.W. Kim, P.; Muja, N.; Campanelli, J. T.; Bulte, J. W., ICV-transplanted human glial precursor cells are short-lived yet exert immunomodulatory effects in mice with EAE. Glia 60 (2012) 1117-29.

[114] J.H.B. Kim, M. D.; Liang, H. F.; Klein, R. S.; Russell, J. H.; Cross, A. H.; Song, S. K., Detecting axon damage in spinal cord from a mouse model of multiple sclerosis. Neurobiology of Disease 21 (2006) 626-32.

[115] K.S. Kirschbaum, J. K.; Zeller, M. W.; Deumelandt, K.; Bode, J.; Sharma, R.; Krüwel, T.; Fischer, M.; Hoffmann, A.; Da Silva, M. C.; Muckenthaler, M. U.; Wick, W.; Tews, B.; Chen, J. W.; Heiland, S.; Bendszus, M.; Platten, M.; Breckwoldt, M. O., In vivo nanoparticle imaging of innate immune cells can serve as a marker of disease severity in a model of multiple sclerosis. Proceedings of the National Academy of Sciences of the United States of America 113 (2016) 13227-13232.

[116] B.M.D. Krauspe, W.; Beyer, C.; Baumgartner, W.; Denecke, B.; Janssen, K.; Langhans, C. D.; Clarner, T.; Kipp, M., Short-term cuprizone feeding verifies N-acetylaspartate quantification as a marker of neurodegeneration. Journal of Molecular Neuroscience 55 (2015) 733-48.

[117] M.A.E. Kuharik, M. K.; Farlow, M. R.; Becker, G. J.; Azzarelli, B.; Klatte, E. C.; Augustyn, G. T.; Dreesen, R. G., Gd-enhanced MR imaging of acute and chronic experimental demyelinating lesions. AJNR Am J Neuroradiol 9 (1988) 643-8.

[118] G.J. Ladewig, L.; Misselwitz, B.; Solymosi, L.; Toyka, K.; Bendszus, M.; Stoll, G., Spatial diversity of blood-brain barrier alteration and macrophage invasion in experimental autoimmune encephalomyelitis: a comparative MRI study. Experimental Neurology 220 (2009) 207-11.

[119] J.D.t.H. Laman, B. A.; Brok, H.; Meurs, Mv; Schellekens, M. M.; Kasran, A.; Boon, L.; Bauer, J.; Boer, Md; Ceuppens, J., Protection of marmoset monkeys against EAE by treatment with a murine antibody blocking CD40 (mu5D12). European Journal of Immunology 32 (2002) 2218-28.

[120] D.G. Le Blon, C.; Hoornaert, C.; Quarta, A.; Daans, J.; Dooley, D.; Lemmens, E.; Praet, J.; De Vocht, N.; Reekmans, K.; Santermans, E.; Hens, N.; Goossens, H.; Verhoye, M.; Van der Linden, A.; Berneman, Z.; Hendrix, S.; Ponsaerts, P., Intracerebral transplantation of interleukin 13-producing mesenchymal stem cells limits microgliosis, oligodendrocyte loss and demyelination in the cuprizone mouse model. Journal of Neuroinflammation 13 (2016) 288.

[121] J.S. Lee, K.; Kang, B. T.; Yao, B.; Fukunaga, M.; van Gelderen, P.; Palumbo, S.; Bosetti, F.; Silva, A. C.; Duyn, J. H., The contribution of myelin to magnetic susceptibility-weighted contrasts in high-field MRI of the brain. NeuroImage 59 (2012) 3967-75.

[122] H.B. Levy Barazany, D.; Puckett, L.; Blanga-Kanfi, S.; Borenstein-Auerbach, N.; Yang, K.; Peron, J. P.; Weiner, H. L.; Frenkel, D., Brain MRI of nasal MOG therapeutic effect in relapsing-progressive EAE. Experimental Neurology 255 (2014) 63-70.

[123] H.A. Levy, Y.; Frenkel, D., Characterization of brain lesions in a mouse model of progressive multiple sclerosis. Experimental Neurology 226 (2010) 148-58.

[124] Y.Z. Li, Y.; Han, W.; Hu, F.; Qian, Y.; Chen, Q., TRO19622 promotes myelin repair in a rat model of demyelination. International Journal of Neuroscience 123 (2013) 810-22.

[125] K.S. Li-ChunHsieh, S.; Zeller, M. W. G.; Pulli, B.; Ali, M.; Wang, C. H.; Chiou, T. T. Y.; Tsang, Y. M.; Lee, P. S.; Stossel, T. P.; Chen, J. W., Gelsolin decreases actin toxicity and inflammation in murine multiple sclerosis. Journal of neuroimmunology 287 (2015) 36-42.

[126] T.H.K. Lin, J. H.; Perez-Torres, C.; Chiang, C. W.; Trinkaus, K.; Cross, A. H.; Song, S. K., Axonal transport rate decreased at the onset of optic neuritis in EAE mice. NeuroImage 100 (2014) 244-53.

[127] T.H.S. Lin, W. M.; Chiang, C. W.; Trinkaus, K.; Cross, A. H.; Song, S. K., Diffusion fMRI detects white-matter dysfunction in mice with acute optic neuritis. Neurobiology of Disease 67 (2014) 1-8.

[128] R.A.K. Linker, A.; Horn, T.; Gold, R.; Maurer, M.; Bendszus, M., Iron particle-enhanced visualization of inflammatory central nervous system lesions by high resolution: preliminary data in an animal model. AJNR Am J Neuroradiol 27 (2006) 1225-9.

[129] R.A.R. Linker, M.; Bendszus, M.; Ladewig, G.; Briel, A.; Schirner, M.; Maurer, M.; Hauff, P., In vivo molecular imaging of adhesion molecules in experimental autoimmune encephalomyelitis (EAE). Journal of Autoimmunity 25 (2005) 199-205.

[130] G.A.W. Lodygensky, T.; Stump, M.; Holtzman, D. M.; Inder, T. E.; Neil, J. J., In vivo MRI analysis of an inflammatory injury in the developing brain. Brain Behav Immun 24 (2010) 759-67.

[131] A.M. Luchetti, D.; Ruffini, F.; Galli, R.; Falini, A.; Quattrini, A.; Scotti, G.; Comi, G.; Martino, G.; Furlan, R.; Politi, L. S., Monoclonal antibodies conjugated with superparamagnetic iron oxide particles allow magnetic resonance imaging detection of lymphocytes in the mouse brain. Molecular Imaging 11 (2012) 114-25.

[132] A.R. MacKenzie-Graham, G. A.; Avedisian, A.; Gold, S. M.; Frew, A. J.; Aguilar, C.; Lin, D. R.; Umeda, E.; Voskuhl, R. R.; Alger, J. R., Cortical atrophy in experimental autoimmune encephalomyelitis: in vivo imaging. NeuroImage 60 (2012) 95-104.

[133] A.T. MacKenzie-Graham, M. R.; Shah, K. P.; Aguilar, C.; Strickland, L. V.; Boline, J.; Martin, M.; Morales, L.; Shattuck, D. W.; Jacobs, R. E.; Voskuhl, R. R.; Toga, A. W., Cerebellar cortical atrophy in experimental autoimmune encephalomyelitis. NeuroImage 32 (2006) 1016-23.

[134] A.T.-W. MacKenzie-Graham, S. K.; Sharma, G.; Aguilar, C.; Vo, K. T.; Strickland, L. V.; Morales, L.; Fubara, B.; Martin, M.; Jacobs, R. E.; Johnson, G. A.; Toga, A. W.; Voskuhl, R. R., Purkinje cell loss in experimental autoimmune encephalomyelitis. NeuroImage 48 (2009) 637-51.

[135] K.Z. Magalon, C.; Cayre, M.; Khaldi, J.; Bourbon, C.; Robles, I.; Tardif, G.; Viola, A.; Pruss, R. M.; Bordet, T.; Durbec, P., Olesoxime accelerates myelination and promotes repair in models of demyelination. Annals of Neurology 71 (2012) 213-26.

[136] P.M. Maggi, S. M. C.; Gaitán, M. I.; Leibovitch, E.; Wholer, J. E.; Knight, H. L.; Ellis, M.; Wu, T.; Silva, A. C.; Massacesi, L.; Jacobson, S.; Westmoreland, S.; Reich, D. S., The formation of inflammatory demyelinated lesions in cerebral white matter. Annals of Neurology (2014).

[137] S.S. Mardiguian, S.; Ladds, E.; Campbell, S. J.; Wilainam, P.; McFadyen, C.; McAteer, M.; Choudhury, R. P.; Smith, P.; Saunders, F.; Watt, G.; Sibson, N. R.; Anthony, D. C., Anti-IL-17A treatment reduces clinical score and VCAM-1 expression detected by in vivo magnetic resonance imaging in chronic relapsing EAE ABH mice. American Journal of Pathology 182 (2013) 2071-81.

[138] M.P.E. Marriott, B.; Cate, H. S.; Binder, M. D.; Kemper, D.; Wu, Q.; Kolbe, S.; Gordon, I. R.; Wang, H.; Egan, G.; Murray, S.; Butzkueven, H.; Kilpatrick, T. J., Leukemia inhibitory factor signaling modulates both central nervous system demyelination and myelin repair. Glia 56 (2008) 686-98.

[139] M.A.S. McAteer, N. R.; von Zur Muhlen, C.; Schneider, J. E.; Lowe, A. S.; Warrick, N.; Channon, K. M.; Anthony, D. C.; Choudhury, R. P., In vivo magnetic resonance imaging of acute brain inflammation using microparticles of iron oxide. Nature Medicine 13 (2007) 1253-8.

[140] M.A.v.Z.M. McAteer, C.; Anthony, D. C.; Sibson, N. R.; Choudhury, R. P., Magnetic Resonance Imaging of Brain Inflammation Using Microparticles of Iron Oxide. in: K. Shah, (Ed.), Moleuclar Imaging: Methods and Protocols, Humana Press Inc, Totowa, 2011, pp. 103-115.

[141] C.R.B. McCreary, T. A.; Skihar, V.; Mitchell, J. R.; Yong, V. W.; Dunn, J. F., Multiexponential T2 and magnetization transfer MRI of demyelination and remyelination in murine spinal cord. NeuroImage 45 (2009) 1173-82.

[142] D.B. Merkler, S.; Stadelmann, C.; Ernsting, T.; Michaelis, T.; Frahm, J.; Bruck, W., Multicontrast MRI of remyelination in the central nervous system. NMR in biomedicine 18 (2005) 395-403.

[143] S.H. Mi, B.; Hahm, K.; Luo, Y.; Kam Hui, E. S.; Yuan, Q.; Wong, W. M.; Wang, L.; Su, H.; Chu, T. H.; Guo, J.; Zhang, W.; So, K. F.; Pepinsky, B.; Shao, Z.; Graff, C.; Garber, E.; Jung, V.; Wu, E. X.; Wu, W., LINGO-1 antagonist promotes spinal cord remyelination and axonal integrity in MOG-induced experimental autoimmune encephalomyelitis. Nature Medicine 13 (2007) 1228-33.

[144] J.D.-C. Mikita, N.; Deloire, M. S.; Vekris, A.; Biran, M.; Raffard, G.; Brochet, B.; Canron, M. H.; Franconi, J. M.; Boiziau, C.; Petry, K. G., Altered M1/M2 activation patterns of monocytes in severe relapsing experimental rat model of multiple sclerosis. Amelioration of clinical status by M2 activated monocyte administration. Multiple Sclerosis 17 (2011) 2-15.

[145] J.M.S. Millward, J.; Taupitz, M.; Wagner, S.; Wuerfel, J. T.; Infante-Duarte, C., Iron oxide magnetic nanoparticles highlight early involvement of the choroid plexus in central nervous system inflammation. ASN Neuro 5 (2013) e00110.

[146] J.H.J. Moon, H. W.; Lee, H. C.; Jeon, J. H.; Kim, N. H.; Sur, J. H.; Ha, J.; Jung, D. I., A study of experimental autoimmune encephalomyelitis in dogs as a disease model for canine necrotizing encephalitis. J vet sci 16 (2015) 203-11.

[147] S.P.D. Morrissey, R.; Syha, J.; Simonis, C.; Zettl, U.; Archelos, J. J.; Jung, S.; Stodal, H.; Lassmann, H.; Toyka, K. V.; Haase, A.; Hartung, H. P., Partial inhibition of AT-EAE by an antibody to ICAM-1: clinico-histological and MRI studies. Journal of neuroimmunology 69 (1996) 85-93.

[148] S.P.S. Morrissey, H.; Zettl, U.; Simonis, C.; Jung, S.; Kiefer, R.; Lassmann, H.; Hartung, H. P.; Haase, A.; Toyka, K. V., In vivo MRI and its histological correlates in acute adoptive transfer experimental allergic encephalomyelitis. Quantification of inflammation and oedema. Brain 119 (1996) 239-48.

[149] N.C. Muja, M. E.; Zhang, J.; Kim, H.; Gilad, A. A.; Walczak, P.; Ben-Hur, T.; Bulte, J. W., Neural precursors exhibit distinctly different patterns of cell migration upon transplantation during either the acute or chronic phase of EAE: a serial MR imaging study. Magnetic Resonance in Medicine 65 (2011) 1738-49.

[150] I.J.S. Namaer, J.; Poulet, P.; Armspach, J. P.; Mauss, Y.; Chambron, J., In vivo dynamic MR imaging of MBP-induced acute experimental allergic encephalomyelitis in Lewis rat. Magnetic Resonance in Medicine 24 (1992) 325-334.

[151] I.J.S. Namer, J.; Poulet, P.; Armspach, J. P.; Mohr, M.; Mauss, Y.; Chambron, J., Blood-brain barrier breakdown in MBP-specific T cell induced experimental allergic encephalomyelitis. A quantitative in vivo MRI study. Brain 116 (1993) 147-59.

[152] I.J.S. Namer, J.; Piddlesden, S. J.; Mohr, M.; Poulet, P.; Chambron, J., Magnetic resonance imaging of antibody-mediated demyelinating experimental allergic encephalomyelitis. Journal of neuroimmunology 54 (1994) 41-50.

[153] I.J.S. Namer, J.; Poulet, P.; Mauss, Y.; Armspach, J. P.; Eclancher, B.; Chambron, J., Hyperbaric oxygen treatment in acute experimental allergic encephalomyelitis. Contribution of magnetic resonance imaging study. NeuroImage 1 (1994) 308-12.

[154] I.J.S. Namer, J.; Klinguer, C.; Trifilieff, E.; Mohr, M.; Poulet, P., Magnetic resonance imaging of PLP-induced experimental allergic encephalomyelitis in Lewis rats. Journal of neuroimmunology 92 (1998) 22-8.

[155] N.A. Nathoo, S.; Wu, Y.; Haylock-Jacobs, S.; Yong, V. W.; Foniok, T.; Barnes, S.; Obenaus, A.; Dunn, J. F., Susceptibility-weighted imaging in the experimental autoimmune encephalomyelitis model of multiple sclerosis indicates elevated deoxyhemoglobin, iron deposition and demyelination. Multiple Sclerosis 19 (2013) 721-31.

[156] N.R. Nathoo, J. A.; Yong, V. W.; Dunn, J. F., Detecting deoxyhemoglobin in spinal cord vasculature of the experimental autoimmune encephalomyelitis mouse model of multiple sclerosis using susceptibility MRI and hyperoxygenation. PLoS ONE 10 (2015) e0127033.

[157] S.B. Nessler, S.; Stadelmann, C.; Bittner, A.; Merkler, D.; Hartung, H. P.; Michaelis, T.; Bruck, W.; Frahm, J.; Sommer, N.; Hemmer, B., Early MRI changes in a mouse model of multiple sclerosis are predictive of severe inflammatory tissue damage. Brain 130 (2007) 2186-98.

[158] T.T.Y. Nie, G.; Jia, Y. L.; Zhang, T.; Shen, Z. W.; Zhang, H. D.; Xu, H. Y.; Wu, R. H., Region-Specific Susceptibilities to Cuprizone-Induced Demyelination of C57BL/6 Mouse: In vivo T2WI and DTI Studies at 7.0T. Appl. Magn. Reson. 45 (2014) 759-769.

[159] J.H.G. Noseworthy, J. J.; Vandervoort, M. K.; Karlik, S. J., Postnatal NMR changes in guinea pig central nervous system: potential relevance to experimental allergic encephalomyelitis. Magnetic Resonance in Medicine 6 (1988) 199-211.

[160] U.M. Noth, S. P.; Deichmann, R.; Jung, S.; Adolf, H.; Haase, A.; Lutz, J., Perfluoro-15-crown-5-ether labelled macrophages in adoptive transfer experimental allergic encephalomyelitis. Artif Cells Blood Substit Immobil Biotechnol 25 (1997) 243-54.

[161] J.K. Orije, F.; Guglielmetti, C.; Praet, J.; Van der Linden, A.; Ponsaerts, P.; Verhoye, M., Longitudinal monitoring of metabolic alterations in cuprizone mouse model of multiple sclerosis using 1H-magnetic resonance spectroscopy. NeuroImage 114 (2015) 128-35.

[162] R.D.B. Oude Engberink, E. L.; Dijkstra, C. D.; van der Pol, S. M.; van der Toorn, A.; de Vries, H. E., Dynamics and fate of USPIO in the central nervous system in experimental autoimmune encephalomyelitis. NMR in biomedicine 23 (2010) 1087-96.

[163] A.J.D. Oweida, E. A.; Foster, P. J., Cellular imaging at 1.5 T: detecting cells in neuroinflammation using active labeling with superparamagnetic iron oxide. Molecular Imaging 3 (2004) 85-95.

[164] A.J.D. Oweida, E. A.; Karlik, S. J.; Dekaban, G. A.; Foster, P. J., Iron-oxide labeling of hematogenous macrophages in a model of experimental autoimmune encephalomyelitis and the contribution to

signal loss in fast imaging employing steady state acquisition (FIESTA) images. Journal of Magnetic Resonance Imaging 26 (2007) 144-51.

[165] J.D. Palazuelos, N.; Julien, B.; Hatterer, E.; Aguado, T.; Mechoulam, R.; Benito, C.; Romero, J.; Silva, A.; Guzmán, M.; Nataf, S.; Galve-Roperh, I., The CB2 cannabinoid receptor controls myeloid progenitor trafficking: Involvement in the pathogenesis of an animal model of multiple sclerosis. Journal of Biological Chemistry 283 (2008) 13320-13324.

[166] M.M.R. Paz Soldan, M. R.; Gamez, J. D.; Lohrey, A. K.; Chen, Y.; Pirko, I.; Johnson, A. J., Correlation of Brain Atrophy, Disability, and Spinal Cord Atrophy in a Murine Model of Multiple Sclerosis. Journal of Neuroimaging 25 (2015) 595-9.

[167] G.V.V.d.V. Peersman, F. L.; Lohman, J. E.; Lubke, U.; Gheuens, J.; Bellon, E.; Connelly, A.; Martin, J. J., High resolution nuclear magnetic resonance imaging of the spinal cord in experimental demyelinating disease. Acta Neuropathologica 76 (1988) 628-32.

[168] A.A. Petiet, M. S.; Stankoff, B., Gray and White Matter Demyelination and Remyelination Detected with Multimodal Quantitative MRI Analysis at 11.7T in a Chronic Mouse Model of Multiple Sclerosis. Frontiers in Neuroscience 10 (2016) 491.

[169] Å.W. Pettersson, X. C.; Ciumas, C.; Lian, H.; Chirsky, V.; Huang, Y. M.; Bjelke, B.; Link, H.; Xiao, B. G., CD8α+ dendritic cells and immune protection from experimental allergic encephalomyelitis. Clinical and Experimental Immunology 137 (2004) 486-495.

[170] P.S.Y. Piraino, T. A.; Freedman, S. B.; Messersmith, E. K.; Pleiss, M. A.; Karlik, S. J., Suppression of acute experimental allergic encephalomyelitis with a small molecule inhibitor of alpha4 integrin. Multiple Sclerosis 11 (2005) 683-90.

[171] I.C. Pirko, B.; Johnson, A. J.; Gamez, J.; Rodriguez, M.; Macura, S., Magnetic resonance imaging of immune cells in inflammation of central nervous system. Croat Med J 44 (2003) 463-8.

[172] I.C. Pirko, B.; Gamez, J.; Bieber, A. J.; Warrington, A. E.; Johnson, A. J.; Hanson, D. P.; Pease, L. R.; Macura, S. I.; Rodriguez, M., A human antibody that promotes remyelination enters the CNS and decreases lesion load as detected by T2-weighted spinal cord MRI in a virus-induced murine model of MS. FASEB Journal 18 (2004) 1577-9.

[173] I.C. Pirko, R.; Chen, Y.; Lohrey, A. K.; Lindquist, D. M.; Dunn, R. S.; Zivadinov, R.; Johnson, A. J., CMV infection attenuates the disease course in a murine model of multiple sclerosis. PLoS ONE 7 (2012) e32767.

[174] I.C. Pirko, Y.; Lohrey, A. K.; McDole, J.; Gamez, J. D.; Allen, K. S.; Pavelko, K. D.; Lindquist, D. M.; Dunn, R. S.; Macura, S. I.; Johnson, A. J., Contrasting roles for CD4 vs. CD8 T-cells in a murine model of virally induced "T1 black hole" formation. PLoS ONE 7 (2012) e31459.

[175] I.G. Pirko, J.; Johnson, A. J.; Macura, S. I.; Rodriguez, M., Dynamics of MRI lesion development in an animal model of viral-induced acute progressive CNS demyelination. NeuroImage 21 (2004) 576-82.

[176] I.J. Pirko, A.; Ciric, B.; Gamez, J.; Macura, S. I.; Pease, L. R.; Rodriguez, M., In vivo magnetic resonance imaging of immune cells in the central nervous system with superparamagnetic antibodies. FASEB Journal 18 (2004) 179-82.

[177] I.J. Pirko, A.; Gamez, J.; Macura, S. I.; Rodriguez, M., Disappearing "T1 black holes" in an animal model of multiple sclerosis. Frontiers in Bioscience 9 (2004) 1222-7.

[178] I.J. Pirko, A. J.; Lohrey, A. K.; Chen, Y.; Ying, J., Deep gray matter T2 hypointensity correlates with disability in a murine model of MS. Journal of the Neurological Sciences 282 (2009) 34-8.

[179] I.J. Pirko, A. J.; Chen, Y.; Lindquist, D. M.; Lohrey, A. K.; Ying, J.; Dunn, R. S., Brain atrophy correlates with functional outcome in a murine model of multiple sclerosis. NeuroImage 54 (2011) 802-6.

[180] L.S.B. Politi, M.; Brambilla, E.; Cadioli, M.; Falini, A.; Comi, G.; Scotti, G.; Martino, G.; Pluchino, S., Magnetic-resonance-based tracking and quantification of intravenously injected neural stem cell accumulation in the brains of mice with experimental multiple sclerosis. Stem Cells 25 (2007) 2583-92.

[181] J.O. Praet, J.; Kara, F.; Guglielmetti, C.; Santermans, E.; Daans, J.; Hens, N.; Verhoye, M.; Berneman, Z.; Ponsaerts, P.; Van der Linden, A., Cuprizone-induced demyelination and demyelination-associated inflammation result in different proton magnetic resonance metabolite spectra. NMR in biomedicine 28 (2015) 505-13.

[182] X.L. Qi, A. S.; Sun, L.; Hauswirth, W. W.; Guy, J., Suppression of mitochondrial oxidative stress provides long-term neuroprotection in experimental optic neuritis. Investigative Ophthalmology & Visual Science 48 (2007) 681-91.

[183] X.S. Qi, L.; Lewin, A. S.; Hauswirth, W. W.; Guy, J., Long-term suppression of neurodegeneration in chronic experimental optic neuritis: antioxidant gene therapy. Investigative Ophthalmology & Visual Science 48 (2007) 5360-70.

[184] M.H. Rausch, P.; Baumann, D.; Cannet, C.; Rudin, M., MRI-based monitoring of inflammation and tissue damage in acute and chronic relapsing EAE. Magnetic Resonance in Medicine 50 (2003) 309-14.

[185] M.H. Rausch, P.; Foster, C. A.; Baumann, D. R.; Cannet, C.; Rudin, M., Predictability of FTY720 efficacy in experimental autoimmune encephalomyelitis by in vivo macrophage tracking: clinical implications for ultrasmall superparamagnetic iron oxide-enhanced magnetic resonance imaging. Journal of Magnetic Resonance Imaging 20 (2004) 16-24.

[186] M.T. Rausch, P.; Lervik, P.; Walmsley, A.; Mir, A.; Schubart, A.; Seabrook, T., Characterization of white matter damage in animal models of multiple sclerosis by magnetization transfer ratio and quantitative mapping of the apparent bound proton fraction f. Multiple Sclerosis 15 (2009) 16-27.

[187] T.L.A. Richards, E. C., Jr.; Peterson, J.; Cosgrove, S.; Petersen, R.; Petersen, K.; Heide, A. C.; Cluff, J.; Rose, L. M., Experimental allergic encephalomyelitis in non-human primates: MRI and MRS may predict the type of brain damage. NMR in biomedicine 8 (1995) 49-58.

[188] K.M.N. Robinson, J. M.; Phillips, D. A.; Proctor, T. M.; Rooney, W. D.; Jones, R. E., MR imaging of inflammation during myelin-specific T cell-mediated autoimmune attack in the EAE mouse spinal cord. Mol Imaging Biol 12 (2010) 240-9.

[189] L.M.R. Rose, T. L.; Alvord, E. C., Jr., Magnetic resonance imaging and peripheral blood abnormalities in experimental allergic encephalomyelitis. Biomed Pharmacother 43 (1989) 347-53.

[190] L.M.R. Rose, T. L.; Peterson, J.; Petersen, R.; Alvord, E. C., Jr., Resolution of CNS lesions following treatment of experimental allergic encephalomyelitis in macaques with monoclonal antibody to the CD18 leukocyte integrin. Multiple Sclerosis 2 (1997) 259-66.

[191] N.M. Saskia Hübner, A. E.; Lee, H. L.; Reisert, M.; Bienert, T.; Hennig, J.; von Elverfeldt, D.; Harsan, L. A., The connectomics of brain demyelination: Functional and structural patterns in the cuprizone mouse model. NeuroImage 146 (2017) 1-18.

[192] A.E.B. Schellenberg, R.; Yong, V. W.; Del Bigio, M. R.; Peeling, J., Magnetic resonance imaging of blood-spinal cord barrier disruption in mice with experimental autoimmune encephalomyelitis. Magnetic Resonance in Medicine 58 (2007) 298-305.

[193] C.S. Schneider, G.; Zollner, T. M., Acute neuroinflammation in Lewis rats - a model for acute multiple sclerosis relapses. Journal of neuroimmunology 213 (2009) 84-90.

[194] P.A.S. Seeldrayers, J.; Morrissey, S. P.; Stodal, H.; Vass, K.; Jung, S.; Gneiting, T.; Lassmann, H.; Haase, A.; Hartung, H. P.; et al.,, Magnetic resonance imaging investigation of blood-brain barrier damage in adoptive transfer experimental autoimmune encephalomyelitis. Journal of neuroimmunology 46 (1993) 199-206.

[195] S.A. Serres, D. C.; Jiang, Y.; Broom, K. A.; Campbell, S. J.; Tyler, D. J.; van Kasteren, S. I.; Davis, B. G.; Sibson, N. R., Systemic inflammatory response reactivates immune-mediated lesions in rat brain. Journal of Neuroscience 29 (2009) 4820-8.

[196] S.B. Serres, C.; de Pablos, R. M.; Merkler, D.; Soto, M. S.; Sibson, N. R.; Anthony, D. C., Magnetic resonance imaging reveals therapeutic effects of interferon-beta on cytokine-induced reactivation of rat model of multiple sclerosis. J Cereb Blood Flow Metab 33 (2013) 744-53.

[197] S.M. Serres, S.; Campbell, S. J.; McAteer, M. A.; Akhtar, A.; Krapitchev, A.; Choudhury, R. P.; Anthony, D. C.; Sibson, N. R., VCAM-1-targeted magnetic resonance imaging reveals subclinical disease in a mouse model of multiple sclerosis. FASEB Journal 25 (2011) 4415-22.

[198] N.R.B. Sibson, A. M.; Bernades-Silva, M.; Laurent, S.; Boutry, S.; Muller, R. N.; Styles, P.; Anthony, D. C., MRI detection of early endothelial activation in brain inflammation. Magnetic Resonance in Medicine 51 (2004) 248-52.

[199] B.A.T. Singer, N. J.; Frank, J. A.; McFarland, H. F.; Biddison, W. E., Induction of experimental allergic encephalomyelitis in the NIH minipig. Journal of neuroimmunology 105 (2000) 7-19.

[200] D.A.G. Sipkins, K.; Tropper, F. D.; Bednarski, M.; Li, K. C. P.; Steinman, L., ICAM-1 expression in autoimmune encephalitis visualized using magnetic resonance imaging. Journal of neuroimmunology 104 (2000) 1-9.

[201] S.K.Y. Song, J.; Le, T. Q.; Lin, S. J.; Sun, S. W.; Cross, A. H.; Armstrong, R. C., Demyelination increases radial diffusivity in corpus callosum of mouse brain. NeuroImage 26 (2005) 132-40.

[202] R.M.H. Stassart, G.; Garea-Rodríguez, E.; Nessler, S.; Hayardeny, L.; Wegner, C.; Schlumbohm, C.; Fuchs, E.; Brück, W., A New Targeted Model of Experimental Autoimmune Encephalomyelitis in the Common Marmoset. Brain Pathology 26 (2016) 452-464.

[203] A.W. Steinbrecher, T.; Neuberger, T.; Mueller, A. M.; Pedre, X.; Giegerich, G.; Bogdahn, U.; Jakob, P.; Haase, A.; Faber, C., Experimental autoimmune encephalomyelitis in the rat spinal cord: lesion detection with high-resolution MR microscopy at 17.6 T. AJNR Am J Neuroradiol 26 (2005) 19-25.

[204] W.A.A. Stewart, E. C.; Hruby, S.; Hall, L. D.; Paty, D. W., Early detection of experimental allergic encephalomyelitis by magnetic resonance imaging. Lancet 2 (1985) 898.

[205] W.A.A. Stewart, E. C., Jr.; Hruby, S.; Hall, L. D.; Paty, D. W., Magnetic resonance imaging of experimental allergic encephalomyelitis in primates. Brain 114 (1991) 1069-96.

[206] G.W. Stoll, C.; Gold, R.; Solymosi, L.; Toyka, K. V.; Bendszus, M., In vivo monitoring of macrophage infiltration in experimental autoimmune neuritis by magnetic resonance imaging. Journal of neuroimmunology 149 (2004) 142-6.

[207] S.W.L. Sun, H. F.; Trinkaus, K.; Cross, A. H.; Armstrong, R. C.; Song, S. K., Noninvasive detection of cuprizone induced axonal damage and demyelination in the mouse corpus callosum. Magnetic Resonance in Medicine 55 (2006) 302-8.

[208] S.W.L. Sun, H. F.; Schmidt, R. E.; Cross, A. H.; Song, S. K., Selective vulnerability of cerebral white matter in a murine model of multiple sclerosis detected using diffusion tensor imaging. Neurobiology of Disease 28 (2007) 30-8.

[209] B.A.B. T Hart, J.; Muller, H. J.; Melchers, B.; Nicolay, K.; Brok, H.; Bontrop, R. E.; Lassmann, H.; Massacesi, L., Histopathological characterization of magnetic resonance imaging- detectable brain white matter lesions in a primate model of multiple sclerosis: A correlative study in the experimental autoimmune encephalomyelitis model in common marmosets (Callithrix jacchus). American Journal of Pathology 153 (1998) 649-663.

[210] B.A.B. t Hart, E. L.; Brok, H. P.; Boon, L.; de Boer, M.; Bauer, J.; Laman, J. D., Treatment with chimeric anti-human CD40 antibody suppresses MRI-detectable inflammation and enlargement of pre-existing brain lesions in common marmosets affected by MOG-induced EAE. Journal of neuroimmunology 163 (2005) 31-9.

[211] B.A.B. t Hart, H. P.; Remarque, E.; Benson, J.; Treacy, G.; Amor, S.; Hintzen, R. Q.; Laman, J. D.; Bauer, J.; Blezer, E. L., Suppression of ongoing disease in a nonhuman primate model of multiple sclerosis by a human-anti-human IL-12p40 antibody. Journal of Immunology 175 (2005) 4761-8.

[212] I.O.C. Tagge, A.; Chaudhary, P.; Pollaro, J.; Berlow, Y.; Chalupsky, M.; Bourdette, D.; Woltjer, R.; Johnson, M.; Rooney, W., Spatio-Temporal Patterns of Demyelination and Remyelination in the Cuprizone Mouse Model. PLoS ONE 11 (2016) e0152480.

[213] J.F.N.-L. Talbott, Y. S.; Wendland, M. F.; Mukherjee, P.; Huie, J. R.; Hess, C. P.; Mabray, M. C.; Bresnahan, J. C.; Beattie, M. S., Diffusion-Weighted Magnetic Resonance Imaging Characterization of White Matter Injury Produced by Axon-Sparing Demyelination and Severe Contusion Spinal Cord Injury in Rats. Journal of neurotrauma 33 (2016) 929-42.

[214] V.Y. Talla, C.; Shaw, G.; Porciatti, V.; Koilkonda, R. D.; Guy, J., Noninvasive assessments of optic nerve neurodegeneration in transgenic mice with isolated optic neuritis. Investigative Ophthalmology & Visual Science 54 (2013) 4440-50.

[215] S.P.-J. Tambalo, L.; Rigolio, R.; Fiorini, S.; Bontempi, P.; Mallucci, G.; Balzarotti, B.; Marmiroli, P.; Sbarbati, A.; Cavaletti, G.; Pluchino, S.; Marzola, P., Functional Magnetic Resonance Imaging of Rats with Experimental Autoimmune Encephalomyelitis Reveals Brain Cortex Remodeling. Journal of Neuroscience 35 (2015) 10088-100.

[216] J.D.Z. Thiessen, Y.; Zhang, H.; Wang, L.; Buist, R.; Del Bigio, M. R.; Kong, J.; Li, X. M.; Martin, M., Quantitative MRI and ultrastructural examination of the cuprizone mouse model of demyelination. NMR in biomedicine 26 (2013) 1562-81.

[217] J.E.X. Tobin, M.; Le, T. Q.; Song, S. K.; Armstrong, R. C., Reduced axonopathy and enhanced remyelination after chronic demyelination in fibroblast growth factor 2 (Fgf2)-null mice: differential detection with diffusion tensor imaging. J Neuropathol Exp Neurol 70 (2011) 157-65.

[218] O.B. Torkildsen, L. A.; Thorsen, F.; Mork, S. J.; Stangel, M.; Myhr, K. M.; Bo, L., Effects of dietary intervention on MRI activity, de- and remyelination in the cuprizone model for demyelination. Experimental Neurology 215 (2009) 160-6.

[219] T.H. Tourdias, B.; Raffard, G.; Biran, M.; Nishiguchi, T.; Aussudre, J.; Franconi, J. M.; Brochet, B.; Petry, K. G.; Dousset, V., Adapted focal experimental autoimmune encephalomyelitis to allow MRI exploration of multiple sclerosis features. Experimental Neurology 230 (2011) 248-57.

[220] T.M. Tourdias, N.; Dragonu, I.; Cassagno, N.; Boiziau, C.; Aussudre, J.; Brochet, B.; Moonen, C.; Petry, K. G.; Dousset, V., Differential aquaporin 4 expression during edema build-up and resolution phases of brain inflammation. Journal of Neuroinflammation 8 (2011) 143.

[221] L.M. Turati, M.; Mastropietro, A.; Dowell, N. G.; Zucca, I.; Erbetta, A.; Cordiglieri, C.; Brenna, G.; Bianchi, B.; Mantegazza, R.; Cercignani, M.; Baggi, F.; Minati, L., In vivo quantitative magnetization transfer imaging correlates with histology during de- and remyelination in cuprizone-treated mice. NMR in biomedicine 28 (2015) 327-37.

[222] E.A. Tysiak, P.; Aktas, O.; Waiczies, H.; Smyth, M.; Schnorr, J.; Taupitz, M.; Wuerfel, J., Beyond blood brain barrier breakdown - in vivo detection of occult neuroinflammatory foci by magnetic nanoparticles in high field MRI. Journal of Neuroinflammation 6 (2009) 20.

[223] R.J. Van Lambalgen, M., Experimental allergic encephalomyelitis in rhesus monkeys: II. Treatment of EAE with anti-T lymphocyte subset monoclonal antibodies. Clin Exp Immunol 68 (1987) 305-12.

[224] M.R.G. Verhoye, E. J.; Raman, E. R.; Van Reempts, J.; Van der Linden, A., In vivo noninvasive determination of abnormal water diffusion in the rat brain studied in an animal model for multiple sclerosis by diffusion-weighted NMR imaging. Magnetic Resonance Imaging 14 (1996) 521-32.

[225] E.R. Vowinckel, D.; Becher, B.; Verge, G.; Evans, A.; Owens, T.; Antel, J. P., PK11195 binding to the peripheral benzodiazepine receptor as a marker of microglia activation in multiple sclerosis and experimental autoimmune encephalomyelitis. Journal of Neuroscience Research 50 (1997) 345-53.

[226] H.L. Waiczies, S.; Drechsler, S.; Qadri, F.; Purfurst, B.; Sydow, K.; Dathe, M.; Kuhne, A.; Lindel, T.; Hoffmann, W.; Pohlmann, A.; Niendorf, T.; Waiczies, S., Visualizing brain inflammation with a shingled-leg radio-frequency head probe for 19F/1H MRI. Scientific Reports 3 (2013) 1280.

[227] H.M. Waiczies, J. M.; Lepore, S.; Infante-Duarte, C.; Pohlmann, A.; Niendorf, T.; Waiczies, S., Identification of cellular infiltrates during early stages of brain inflammation with magnetic resonance microscopy. PLoS ONE 7 (2012) e32796.

[228] X.B. Wang, J. K.; Kim, J. H.; Chen, Y. J.; O'Neal, J.; O'Neil, S. P.; Tu, T. W.; Trinkaus, K.; Song, S. K., Diffusion tensor imaging detects treatment effects of FTY720 in experimental autoimmune encephalomyelitis mice. NMR in biomedicine 26 (2013) 1742-1750.

[229] X.C. Wang, M. F.; Wang, Y.; Sun, P.; Libbey, J. E.; Trinkaus, K.; Fujinami, R. S.; Song, S. K., Diffusion basis spectrum imaging detects and distinguishes coexisting subclinical inflammation, demyelination and axonal injury in experimental autoimmune encephalomyelitis mice. NMR in biomedicine 27 (2014) 843-852.

[230] Y.W. Wang, Q.; Haldar, J. P.; Yeh, F. C.; Xie, M.; Sun, P.; Tu, T. W.; Trinkaus, K.; Klein, R. S.; Cross, A. H.; Song, S. K., Quantification of increased cellularity during inflammatory demyelination. Brain 134 (2011) 3590-601.

[231] R.R. Williams, A. M.; Wang, W. T.; Choi, I. Y.; Lee, P.; Berman, N. E.; Lynch, S. G.; LeVine, S. M., Iron deposition is independent of cellular inflammation in a cerebral model of multiple sclerosis. BMC Neurosci 12 (2011) 59.

[232] T.C.S. Wood, C.; Hurley, S. A.; Vernon, A. C.; Torres, J.; Dell'Acqua, F.; Williams, S. C. R.; Cash, D., Whole-brain ex-vivo quantitative MRI of the cuprizone mouse model. Peerj 2016 (2016).

[233] Q.Z.Y. Wu, Q.; Cate, H. S.; Kemper, D.; Binder, M.; Wang, H. X.; Fang, K.; Quick, M. J.; Marriott, M.; Kilpatrick, T. J.; Egan, G. F., MRI identification of the rostral-caudal pattern of pathology within the corpus callosum in the cuprizone mouse model. Journal of Magnetic Resonance Imaging 27 (2008) 446-53.

[234] E.I.-D. Wuerfel, C.; Glumm, R.; Wuerfel, J. T., Gadofluorine M-enhanced MRI shows involvement of circumventricular organs in neuroinflammation. Journal of Neuroinflammation 7 (2010) 70.

[235] J.T. Wuerfel, E.; Prozorovski, T.; Smyth, M.; Mueller, S.; Schnorr, J.; Taupitz, M.; Zipp, F., Mouse model mimics multiple sclerosis in the clinico-radiological paradox. European Journal of Neuroscience 26 (2007) 190-8.

[236] B.G.W. Xiao, X. C.; Yang, J. S.; Xu, L. Y.; Liu, X.; Huang, Y. M.; Bjelke, B.; Link, H., Therapeutic potential of IFN-gamma-modified dendritic cells in acute and chronic experimental allergic encephalomyelitis. Int Immunol 16 (2004) 13-22.

[237] S.J. Xu, E. K.; Brocke, S.; Bulte, J. W.; Quigley, L.; Tresser, N.; Ostuni, J. L.; Yang, Y.; McFarland, H. F.; Frank, J. A., Study of relapsing remitting experimental allergic encephalomyelitis SJL mouse model using MION-46L enhanced in vivo MRI: early histopathological correlation. Journal of Neuroscience Research 52 (1998) 549-58.

[238] S.J. Xu, E. K.; Li, W.; Yang, Y.; Chesnick, S. A.; Webster, H. D.; Brocke, S.; Quigley, L.; McFarland, H. F.; Frank, J. A., In vivo three-dimensional MR microscopy of mice with chronic relapsing experimental autoimmune encephalomyelitis after treatment with insulin-like growth factor-I. AJNR Am J Neuroradiol 19 (1998) 653-8.

[239] O.S. Yu, J.; Mauss, Y.; Guignard, B.; Eclancher, B.; Chambron, J.; Grucker, D., Remyelination assessment by MRI texture analysis in a cuprizone mouse model. Magnetic Resonance Imaging 22 (2004) 1139-44.

[240] W.D. Zaaraoui, M.; Merle, M.; Girard, C.; Raffard, G.; Biran, M.; Inglese, M.; Petry, K. G.; Gonen, O.; Brochet, B.; Franconi, J. M.; Dousset, V., Monitoring demyelination and remyelination by magnetization transfer imaging in the mouse brain at 9.4 T. Magma 21 (2008) 357-62.

[241] J.J. Zhang, M. V.; McMahon, M. T.; Mori, S.; Calabresi, P. A., In vivo and ex vivo diffusion tensor imaging of cuprizone-induced demyelination in the mouse corpus callosum. Magnetic Resonance in Medicine 67 (2012) 750-759.

[242] Y.W. Zhang, J.; Buist, R.; Peeling, J.; Yong, V. W.; Mitchell, J. R., A novel MRI texture analysis of demyelination and inflammation in relapsing-remitting experimental allergic encephalomyelitis. Med Image Comput Comput Assist Interv Int Conf Med Image Comput Comput Assist Interv 9 (2006) 760-7.

[243] Y.W. Zhang, J.; Buist, R.; Peeling, J.; Yong, V. W.; Mitchell, J. R., Active inflammation increases the heterogeneity of MRI texture in mice with relapsing experimental allergic encephalomyelitis. Magnetic Resonance Imaging 32 (2014) 168-74.

[244] J.N. Zhong, K.; Morel, P. A.; Xu, H.; Ahrens, E. T., In Vivo Quantification of Inflammation in Experimental Autoimmune Encephalomyelitis Rats Using Fluorine-19 Magnetic Resonance Imaging Reveals Immune Cell Recruitment outside the Nervous System. PLoS ONE 10 (2015) e0140238.
