# Supplementary material for: STEED: A data mining tool for automated extraction of experimental parameters and risk of bias items from in vivo publications
Source: PLoS One. 2024 Nov 26;19(11):e0311358. doi: 10.1371/journal.pone.0311358 (PMC11594395; doi:10.1371/journal.pone.0311358)
Supplement: Attachment — Submitted filename: Response_to_reviewers_R1.docx [file pone.0311358.s003.docx]

**Point-by-point response**

Copied from the decision letter, responses in blue.

**Comments made by the Editor**

1. Please ensure that your manuscript meets PLOS ONE's style requirements, including those for file naming. The PLOS ONE style templates can be found at

https://journals.plos.org/plosone/s/file?id=wjVg/PLOSOne_formatting_sample_main_body.pdf and

https://journals.plos.org/plosone/s/file?id=ba62/PLOSOne_formatting_sample_title_authors_affiliations.pdf

Thank you for taking time to edit and review our manuscript.

We formatted our manuscript to meet PloS One’s style requirements.

2. Please note that PLOS ONE has specific guidelines on code sharing for submissions in which author-generated code underpins the findings in the manuscript. In these cases, all author-generated code must be made available without restrictions upon publication of the work. Please review our guidelines at https://journals.plos.org/plosone/s/materials-and-software-sharing#loc-sharing-code and ensure that your code is shared in a way that follows best practice and facilitates reproducibility and reuse.

All custom-made code is freely and fully available at our GitHub page (<https://github.com/Ineichen-Group/Auto-STEED>), as stated in the manuscript (line 100).

3. We note that the grant information you provided in the ‘Funding Information’ and ‘Financial Disclosure’ sections do not match.

When you resubmit, please ensure that you provide the correct grant numbers for the awards you received for your study in the ‘Funding Information’ section.

We will submit the correct funding information.

4. Thank you for stating the following financial disclosure:

"This work was supported by grants of the Swiss National Science Foundation (No. P400PM_183884, to BVI), the UZH Alumni (to BVI), and the Intramural Research Program of NINDS. We thank all our funders for their support."

Please state what role the funders took in the study. If the funders had no role, please state: "The funders had no role in study design, data collection and analysis, decision to publish, or preparation of the manuscript." If this statement is not correct you must amend it as needed.

Please include this amended Role of Funder statement in your cover letter; we will change the online submission form on your behalf.

We added this statement to the cover letter.

5. Please note that funding information should not appear in the Acknowledgments section or other areas of your manuscript. We will only publish funding information present in the Funding Statement section of the online submission form. Please remove any funding-related text from the manuscript.

We removed this section from the manuscript.

6. Please include captions for your Supporting Information files at the end of your manuscript, and update any in-text citations to match accordingly. Please see our Supporting Information guidelines for more information: http://journals.plos.org/plosone/s/supporting-information.

We are now including a caption for our supporting data (i.e., the supplementary reference list) at the end of the manuscript.

7. Please review your reference list to ensure that it is complete and correct. If you have cited papers that have been retracted, please include the rationale for doing so in the manuscript text, or remove these references and replace them with relevant current references. Any changes to the reference list should be mentioned in the rebuttal letter that accompanies your revised manuscript. If you need to cite a retracted article, indicate the article’s retracted status in the References list and also include a citation and full reference for the retraction notice.

We included one additional reference (see below).

8. As the editor and the second reviewer, I agree with the comments made by Reviewer 1. Please ensure that you address each of the specific suggestions in your revised version.

See our comments for R1 below. Thank you again for taking time to edit and review our manuscript.

**Reviewer #1**

Reviewer #1: Thank you for the opportunity to review the manuscript titled “STEED: A data mining tool for automated extraction of experimental parameters and risk of bias items from in vivo publications.

The manuscript is concise, and clearly written. I am not a programmer but rather a potential “user” of the developed R package. Nevertheless, I found the writing to be easy to follow. The authors are open about the potential advantages as well as limitations of their software.

The supplementary materials are very helpful, especially the reporting table to give readers an idea of what the end product looks like. I have very little in terms of criticism.

L 26 In the abstract, you might want to consider using “feasibility” instead of “applicability”. Systematic reviews are still applicable, especially when there is a large body of literature, but they may not be feasible to undertake because of the labor involved.

Thank you for reviewing our manuscript and for the positive and constructive comments.

We agree with this suggestion and adjusted it accordingly.

In the discussion section, could you comment on the potential to adapt the package to other topics? Do you foresee separate packets to be developed for each discipline through adaptation of the regular expressions?

This is an important point you raise. We amended the first paragraph of the discussion to discuss this in more detail which now reads:

“STEED performed well on literature corpora outside of the field is has been developed in as well as in corpora with older publication years, i.e., it has been developed in a corpus covering the motor neuron disease literature and performed well in a corpus of the multiple sclerosis literature. Thus, our devel-oped function could be applied to literature bodies of other research fields. However, adapting STEED to new disciplines requires some consideration: While the tool has shown flexibility across related fields, creating discipline-specific versions may necessitate refining the underlying RegExes to accurately capture more distinct experimental parameters pertinent to each field. This process would involve collaborative efforts with domain experts to ensure the tool's precision and subsequent valida-tion [19]. Consequently, while separate packages for each discipline are conceivable, they would re-quire some adaptation efforts to maintain STEED's standards of accuracy and utility.” (Page 14).

We also added this reference:

19.	Mohammadi E, Karami A. Exploring research trends in big data across disciplines: A text mining analysis. Journal of Information Science. 2022;48(1):44-56.

I agree with the authors that the tool may be able to at least eliminate one reviewer, while not quite replace all human reviewers yet.

Well done!

Again, thank you for taking time to review our manuscript.
